# An Autonomous Log Storage Management Protocol with Blockchain Mechanism and Access Control for the Internet of Things

**DOI:** 10.3390/s20226471

**Published:** 2020-11-12

**Authors:** Chien-Lung Hsu, Wei-Xin Chen, Tuan-Vinh Le

**Affiliations:** 1 Department Information Management, Chang Gung University, Taoyuan 33302, Taiwan; codychen0704@gmail.com; 2 Graduate Institute of Business and Management, Chang Gung University, Taoyuan 33302, Taiwan; tvle.cgu@gmail.com; 3 Healthy Aging Research Center, Chang Gung University, Taoyuan 33302, Taiwan; 4 Department Visual Communication Design, Ming Chi University of Technology, New Taipei 24301, Taiwan; 5 Department Nursing, Taoyuan Chang Gung Memorial Hospital, Taoyuan 333, Taiwan

**Keywords:** attribute-based access control, digital forensics, evidence legality, sensor log, signature chain

## Abstract

As the Internet of Things (IoT) has become prevalent, a massive number of logs produced by IoT devices are transmitted and processed every day. The logs should contain important contents and private information. Moreover, these logs may be used as evidences for forensic investigations when cyber security incidents occur. However, evidence legality and internal security issues in existing works were not properly addressed. This paper proposes an autonomous log storage management protocol with blockchain mechanism and access control for the IoT. Autonomous model allows sensors to encrypt their logs before sending it to gateway and server, so that the logs are not revealed to the public during communication process. Along with blockchain, we introduce the concept “*signature chain*”. The integration of blockchain and signature chain provides efficient management functions with valuable security properties for the logs, including robust identity verification, data integrity, non-repudiation, data tamper resistance, and the legality. Our work also employs attribute-based encryption to achieve fine-grained access control and data confidentiality. The results of security analysis using AVSIPA toolset, GNY logic and semantic proof indicate that the proposed protocol meets various security requirements. Providing good performance with elliptic curve small key size, short BLS signature, efficient signcryption method, and single sign-on solution, our work is suitable for the IoT.

## 1. Introduction

With the popularization of computers and rapid development of mobile network technologies, Internet of Things (IoT) has become prevalent. Various devices and entities can wirelessly be connected to the internet as long as they are equipped with sensors. Enabled with fifth generation (5G) technology, communication in IoT environments is performed with super low latency, high-peak data rates and massive network capacity [1]. Data aggregation and transmission in IoT networks have been significantly improved, in order to provide better efficiency of energy consumption, network control overhead, delay time, loss packet and aggregation rates [2]. Due to these advances, IoT has huge potentials to change the information technology, enhance reliability of communication systems, as well as improve our life quality. For example, in wireless body area networks (WBAN) [3], sensing data produced by wearable sensors provides rapid diagnostics, efficient treatments and valuable research data. In addition to healthcare [3,4,5], IoT applications have been implemented in a lot of domains, such as energy [6], vehicle [7,8], industrial systems [9], etc.

Logs generated by IoT devices contain important contents and sensitive information. The logs can be stored in cloud systems for convenient management. With the management tools, it is allowed to collect, store, analyze, archive, and dispose of the log information [10]. Specific uses of the logs include device monitoring [11], user behavior analysis [12], or digital forensics [13].

### 1.1. The Problems

Most IoT environments adopt centralized architecture for managing log storage. It suffers from internal threats since the data can be compromised by the management staffs. Moreover, sensitive information of the logs may be revealed to unauthorized persons. The adversary can also tamper with the log for illegal purposes. The integrity of the data needs to be preserved for forensic investigations when security incidents occur [14]. Communicating parties may repudiate data ownership for their own interests or motives, which causes challenges for digital forensics [15]. In addition, the legality of collected evidences must be ensured so that it provides an effective and efficient investigation process. In heterogeneous and distributed IoT environments with various devices and sensors, these concerns become prominent.

For addressing aforesaid problems, it is essential to propose a mechanism which provides integrity, availability, and legality of the logs. Access control to the log data should also be taken into account, which ensures the confidentiality where the log can only be viewed by legitimate parties. Furthermore, the mechanism should bear a rational implementation cost.

### 1.2. Related Works

Blockchain is a secure decentralized database that can track, verify, and safely protect the data from tampering [16]. It provides open and transparent mechanism that does not require third-party intervention. Blockchain has successfully been used in various sectors, such as transportation systems [17], medical record management [18], and so on. The concept of combining IoT and blockchain promotes the quality of data sharing services with automatic workflows [19]. Blockchain was proposed as a security solution for IoT by various works [20,21,22]. The research topics include immutable event logs and data access management [23], sensing data transaction [24], or IoT device authentication [25]. The digital forensics in the IoT architecture can be classified into various layers consisting of cloud forensics, network forensics, and device forensics [26]. As the forensics of massive IoT devices require a lot of resources [27], legal evidences helps in improving investigation efficiency in accordance with the demand of law enforcement agencies [28].

Taguchi et al. [29] proposed a distributed management method for logs using a blockchain scheme. The method provides data tamper resistance and increases access availability. Pourmajidi and Miranskyy [30] introduced Logchain, a blockchain-based log system. Their system can avoid log tampering and provides an immutable platform for the log storage. Hang and Kim [31] designed and implemented blockchain platform for ensuring data integrity of the IoT environments. Hang and Kim focused on the integration and management of IoT data and blockchain mechanism. Whereas, the IoT forensics framework designed by Ryu et al. [13] employed the blockchain to satisfy the requirements of IoT forensics. Their work achieves data tamper proof and non-repudiation in third party-less environments. Persistence and privacy of forensic data were also assured. In their design, specific data produced by IoT devices is written into the block for facilitating evidence collection during digital forensic investigations. Aforesaid works have certain strengths that meet several functionality and security requirements. However, internal confidentiality issue was not addressed since they did not introduce access control mechanism. Moreover, the legality of the evidence preservation in their works was uncertain.

Recently, Li et al. [32] proposed a secure fine-grained data sharing scheme for cloud computing. Even though their scheme provides lightweight computation with access control and forward secrecy, it was not introduced with blockchain mechanism. Zheng et al. [33] introduced a new attribute-based encryption scheme using blockchain technique. Their design did not employ digital signature to achieve legal security features. Sowjanya and Dasgupta [34] presented another attribute-based encryption scheme for WBAN. The scheme achieves good performance with elliptic curve cryptography and attribute-based encryption. Zhong et al. [35] also introduced an efficient access control scheme for smart healthcare. Nonetheless, both Sowjanya and Dasgupta [34] and Zhong et al. [35] did not include blockchain mechanism and digital signature technique in their works.

Given the drawbacks of existing works, we are motivated to design a new secure protocol providing log storage management capabilities, fine-grained access control, robust verification, and some other essential security properties. The new design should also meet the forensics requirements as well as the evidence legality.

### 1.3. Main Contributions

Our work proposes an autonomous log storage management protocol with blockchain mechanism and access control for IoT environments. The proposed protocol allows sensor to perform the signcryption of the log data based on its access policy. With access control mechanism, only the authorized users with appropriate attributes are able to unsigncrypt the message and view the log. Each entity in the system has to sign a signature during communication process so that they can be tracked for potential forensics. We integrate blockchain mechanism and digital signature technique to simultaneously achieve various properties. The contributions made in this paper can be described in the following.

- Autonomous model allows sensors to encrypt the logs before sending them to other entities (gateways and servers). Privacy of the logs therefore is fully protected throughout communication process. In this way, our protocol is even secure for communications via unreliable channels. Typical application of this model is WBAN, where wearable sensors encrypt health data before sending it to coordinators and healthcare providers for specific services.
- Since legality of blockchain signature remains uncertain, whereas digital signature satisfies various requirements with legal security properties [36], we introduce the concept “*signature chain*” in this work. A signature chain is composed by the signatures of all communicating entities of the system including sensors, gateway and server. The integration of blockchain and signature chain achieves valuable properties: robust identity verification, data integrity, tamper proof (insider attack resistance), ownership non-repudiation, and evidence legality. Thus, our work is completely helpful to the purposes of digital forensics.
- In our design, private blockchain is employed as a storage to conveniently and efficiently store and process the signature chain and ciphertexts, with various management functions. We adopt Proof of Work (PoW) [37] as the consensus algorithm in proposed private chain, in order to achieve above-mentioned security properties. Due to its full decentralization mechanism and immutability, public blockchain is integrated in our protocol to assure the trust of the private blockchain.
- Fine-grained access control with ciphertext policy attribute-based encryption is proposed in our work. It provides internal confidentiality in which only the legitimate users with specific appropriate attributes are allows to decrypt the ciphertexts and obtain the log plaintexts.
- We use AVISPA toolset and GNY logic to formally prove security correctness of the proposed protocol. Sematic security proof further indicates that our protocol satisfies various security requirements.
- Our work employs elliptic curve with small key size, short BLS signature, and efficient signcryption method to design the protocol with single sign-on solution. Therefore, our protocol bears low computation and storage overhead, which is suitable to the IoT.
- We provide practical implementation of the proposed protocol with specific use case, system construction and user interface.

### 1.4. Paper Structure

The paper is structured as follows. We present preliminaries of our work in Section 2. Section 3.1 provides system model of our work including all entities with communicating roles. Security goals are provided in Section 3.2, which are required for providing a secure communication with the proposed system model. Section 3.3 presents specific procedure and algorithms of the protocol. Section 4 presents security analysis of the proposed protocol including GNY logic, AVISPA toolset, and semantic proof. Performance experiment and analysis of the our protocol are provided in Section 5. Section 6 describes the implementation including practical procedures and system construction of our work. Finally, some concluding remarks and future works are given in Section 7 of the paper.

## 2. Preliminaries

Preliminaries of the paper include linear secret-sharing scheme, attribute-based encryption, signcryption, bilinear map, Boneh-Lynn-Shacham signature, blockchain, and single sign-on.

### 2.1. Linear Secret-Sharing Scheme

Linear Secret-Sharing Scheme (LSSS) proposed by Lewko and Waters [38] introduced how to use AND and OR gates to generate the matrices. LSSS consists of access policy matrix M and column vector v. The matrix M is composed by m rows and n columns, with the policy defined and stored by Boolean formula [39,40]. Whereas, the vector v is composed by $s,a_{1},a_{2},\ldots a_{n}\in_{R}Z_{p}$ that are the randomly selected numbers, in which s is the secret value. Multiplying matrix M with vector v will derive a column vector composed by $\lambda_{1},\ldots \;\lambda_{n}$, where λ is the associated information of the secret value s. Access policy M contains a certain number of attributes. As long as users possess appropriate attributes, they can restore the secret value s.

### 2.2. Attribute-Based Encryption

Attribute-based encryption (ABE) was proposed by Sahai and Waters in 2005 [41]. In ABE, access policy defined by users considers various attributes. The attributes possessed by users determine whether they can meet the policy of data access. This advantage allows an efficient and flexible encryption process. ABE is categorized into two types: key policy attribute-based encryption (KP-ABE) [42,43] and ciphertext policy attribute-based encryption (CP-ABE) [44,45,46]. In the CP-ABE scheme, user’s key is integrated with the attributes; and the ciphertext is associated with the access policy through the LSSS. When access policy is satisfied, the user can use the attribute key to decrypt the ciphertext. On the other hand, in the KP-ABE scheme, the user’s key is associated with the access policy; and the ciphertext is integrated with the attributes. When the ciphertext meets the key’s access policy, the user can decrypt the ciphertext.

### 2.3. Signcryption

Signcryption [47] is the combination of encryption and signature signing. The ciphertext and signature of the message are generated by performing the functions of both encryption and signature at the same time. Compared with the cumulative cost of separate encryption and signing process, this novel method is much more efficient. Signcryption method provides confidentiality, verification and non-repudiation of the given data. Attribute-based signcryption [48] combines the functions of encryption and signature on the attributes. Fine-grained access control can be associated with the signcrypted text to achieve robust message protection. This novel access control mechanism is well suited for data sharing in distributed environments. For example, users outsource their data to cloud storage, and can effectively share the data with other parties. The users who are granted the access can effectively obtain the data from anywhere through the network.

### 2.4. Bilinear Map

Selects a big number q, we have the elliptic curve: $E:y^{2}=x^{3}+ax+b\;mod\;q$. Let $G_{1}$ be a multiplicative cyclic group of order n, and g, $g_{1}$ and $g_{2}$ be the generators of $G_{1}$, a bilinear map from $G_{1}\times G_{1}$ to $G_{T}$ is a function $e:G_{1}\times G_{1}\to G_{T}$. The bilinear map provides the following characteristics and assumption [45,49,50]:

- Bilinear: If any two integers $x,y\in Z_{p}$ and generators $g,g_{1},g_{2}\in G_{1}$, then $e\left(g_{1}^{x},g_{1}^{y}\right)=e{\left(g_{1},g_{1}\right)}^{xy}=e\left(g_{1}^{y},g_{1}^{x}\right),$ and $e\left(g_{1}.g_{2},g\right)=e\left(g_{1},g\right).e\left(g_{2},g\right)$.
- Non-degenerate: There exists $g_{1},g_{2}$ $\in G_{1}$ such that $e\left(g_{1},g_{2}\right)$ is the generator of $G_{T}$.
- Computable: For any $g_{1},g_{2}\in G_{1}$, there exists a polynomial algorithm which can efficiently compute $\;e\left(g_{1},g_{2}\right)$.
- Elliptic Curve Discrete Logarithm Problem (ECDLP): ECDLP is a special case of Discrete Logarithm Problem (DLP), and can be described as follows. Given $g_{1},m\in G_{1}$, the problem is to find integer $x\in Z_{p}$ such that $g_{1}^{x}=m$.

### 2.5. Boneh-Lynn-Shacham Signature Scheme

Boneh-Lynn-Shacham (BLS) scheme [51] provides shorter signature length than Elliptic Curve Digital Signature Algorithm (ECDSA) [52], but with the same security level. BLS signature scheme can provide batch verification function, which allows to sign and verify multiple signatures at once. Given $g_{1},G_{1},G_{T}$ defined in Section 2.4, plaintexts $M_{1}:{\left\{0,1\right\}}^{*}$, $M_{2}:{\left\{0,1\right\}}^{*}$, and hash function $H:{\left\{0,1\right\}}^{*}\in G_{1}$, the procedure of BLS scheme is described as follows:

- Key generation: Randomly choose an integer $x\in_{R}Z_{p}$, let x be private key, we have $Y=g_{1}{}^{x}$ is the corresponding public key.
- Signature generation: Use hash function H and private key x to sign the plaintext $M_{1}$ and generate signature $\sigma_{1}=H{\left(M_{1}\right)}^{x}$.
- Signature verification: Based on plaintext $M_{1}$ and signature $\sigma_{1}$, the verification is to confirm the equation $\left(H\left(M_{1}\right),Y\right)≟e\left(\sigma_{1},g_{1}\right)$. Correctness of the verification is proved as follows: $e\left(H\left(M_{1}\right),Y\right)=e\left(H\left(M_{1}\right),g_{1}{}^{x}\right)=e\left(H{\left(M_{1}\right)}^{x},g_{1}\right)=e\left(\sigma_{1},g_{1}\right)$.
- Batch signature verification: As stated, $\sigma_{1}=H{\left(M_{1}\right)}^{x}$ and $\sigma_{2}=H{\left(M_{2}\right)}^{x}$ are the signatures, the verification is to confirm $e\left(H\left(M_{1}\right)H\left(M_{2}\right),YY\right)≟e\left(\sigma_{1}\sigma_{2},g_{1}g_{1}\right)$. The verification correctness is proved as follows: $e\left(H\left(M_{1}\right)H\left(M_{2}\right),YY\right)=e\left(H\left(M_{1}\right)H\left(M_{2}\right),g_{1}{}^{x}g_{1}{}^{x}\right)=e\left(H{\left(M_{1}\right)}^{x}H{\left(M_{2}\right)}^{x},g_{1}g_{1}\right)=e\left(\sigma_{1}\sigma_{2},g_{1}g_{1}\right)$.

### 2.6. Blockchain

Blockchain was proposed by Nakamoto in 2008 with its first application, Bitcoin [53]. Peer-to-peer (P2P) mechanism of blockchain with distributed ledger is employed to form decentralized networks. Nodes within the networks communicate with each other to confirm the validity of the transactions before they are uploaded to the blockchain. Due to a unique data structure, the content and transaction recorded in blockchain are unalterably protected. Blockchain provides decentralization [54], tamper resistance [55], and user anonymity [56]. There are three types of blockchain: public blockchain, private blockchain and consortium blockchain [57]. In public blockchain, everyone can conduct transactions, verifications and relevant contributions. It is recognized as the concept of completely decentralized open network. Whereas, private blockchain network partly achieves the decentralization since its design allows a single organization to hold central authority. Data access in private blockchain is only granted to a certain number of users based on specific purposes. The consortium blockchain mechanism is similar to the private blockchain. The difference is consortium blockchain includes multiple organizations, which can provide business-to-business (B2B) services.

### 2.7. Single Sign-on

Single Sign-On (SSO) [58] provides multi-server environment that allows users to use a single password to log in multiple servers. After completing identity authentication with one sever, users can freely access the services on other severs within the network, without having to repeat authentication procedure. The benefits of SSO solution can be summarized as follows: (1) Avoids the confusion of users when they must store massive credentials at the same time in single-server environments; (2) Allows central service provider to conveniently manage the authentication information of users; and (3) Significantly reduces credential storage overhead.

## 3. The Proposed Log Storage Management Protocol with Blockchain Mechanism and Access Control

In this section, we describe system model and security goals of the proposed protocol. Thereafter, detailed procedure of our protocol is presented. Cryptographic functions and notations used in the protocol are described in Table 1.

### 3.1. System Model

**Figure 1:**
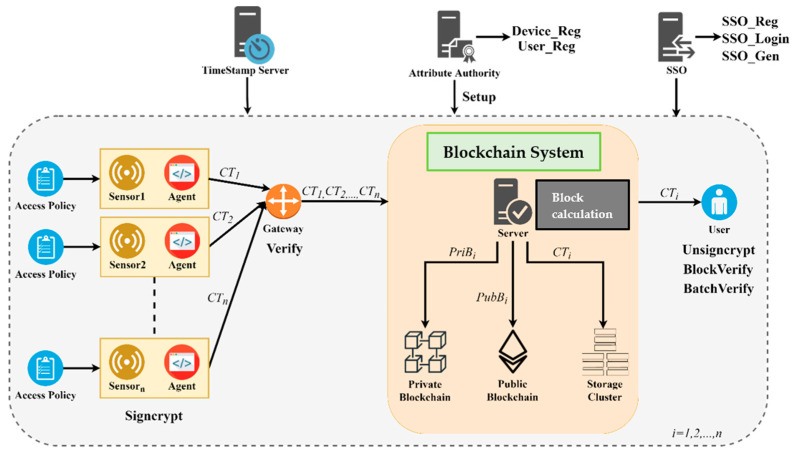
System model of the proposed protocol.

Our system model includes 11 roles: attribute authority, SSO server, timestamp server, sensor (IoT device) and agent, gateway, blockchain server, private blockchain, public blockchain, storage cluster, and user. The attribute authority generates public and secret parameters used in entire communication process. In particular, it sends public parameters to the sensor for log signcryption. The authority also computes private attribute key and transmits it to the user for log unsigncryption. The SSO server provides single sign-on login, allowing users to use a single password to enter multiple servers in multi-server environment. The timestamp server derives timestamp parameters for the system. The sensor is a sensing device which contacts the environment, and generates the logs. The agent is installed inside the sensor, and is responsible for defining the access policy, as well as signcrypting the logs to generate ciphertexts. The gateway verifies the signature included in the ciphertexts to ensure the correctness of the log ciphertext. Blockchain server is responsible for storing the signcrypted text in the storage cluster. Moreover, the server also generates private blocks from single signatures, and public block from multiple signatures, and then writes them into private blockchain and public blockchain respectively. The private blockchain stores signature chains and related information. The public blockchain records corresponding data from the private blockchain, and stores the batch signatures, with fully decentralized nature. The user logs in to the blockchain server through the SSO server, obtains the ciphertext, and uses the attribute private key to unsigncrypt it to view the log plaintext. The user can also verify the validity of the related information stored in private blockchain and public blockchain. System model of the proposed protocol is depicted in Figure 1.

**Figure 2:**
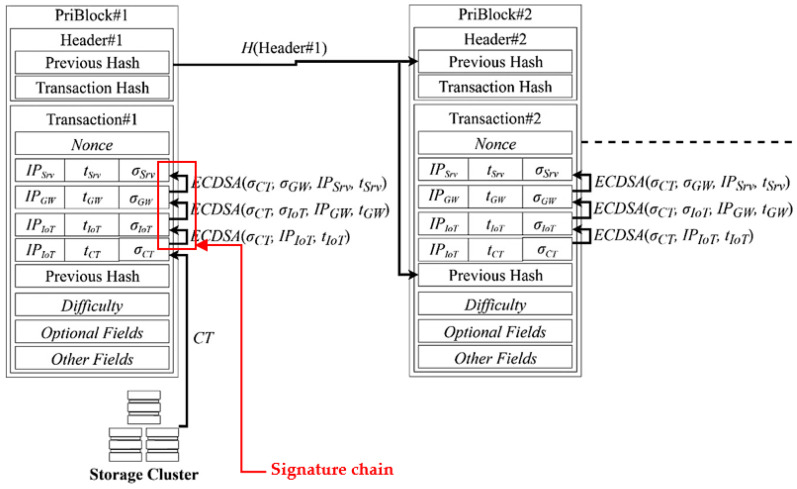
Private blockchain and signature chain in our system model.

Signature chain is composed by the signatures signed by the sensor, the gateway and the server in each communication session. Data in private blockchain is signed using two types of signature schemes including BLS and ECDSA. Each block contains a single signature chain. These chains are immutably stored in blockchain for further security purposes. Figure 2 depicts the design of private blockchain and signature chain of our work.

### 3.2. Security Goals

Security problems are always big concerns in any information systems. The proposed system model includes various parties in a public communication environment. External invasion and security attacks should also be considered for providing a high security environment. We expect that our protocol can satisfy the following security requirements.

- *Secure decryption key*: After the sensor signcrypts the logs, the user attempts to compute the decryption key to decrypt the ciphertext and access the logs. Only legitimate user possessing appropriate attributes is able to compute the correct key.
- *Robust verification*: The digital signature signed by the sensor makes sure that the log data is truly produced and transmitted by the sensor itself. Any parties participating in the communication can verify the validity of the signature.
- *Dataunforgeability*: Only the sensor with its own private key is able to sign the message. We desire to warrant that the signcryption key of the sensor is kept secret to the sensor only, during communication process. In this way, the adversary cannot forge the signature and impersonate the sensor.
- *Datatampering resistance*: The signatures may be modified for obstruction purposes. In addition, the signer may re-sign the message to tamper with its data. These issues should be addressed so that security properties of digital signature are guaranteed.
- *Data confidentiality*: The log data must be kept confidential to the legal parties under any circumstances. Users within the system are allowed to access the logs only if they possess required attributes.
- *Non-repudiation*: Once the logs are signed, signers cannot repudiate them for any own interests. This property is helpful to digital forensic investigations.
- *Data integrity*: This property makes sure that the logs must be originally sent by the sensor without any modifications to its contents.
- *Perfect forward secrecy*: This security goal is required for the long-term decryption key. It ensures that if the adversaries successfully calculate the current decryption key, they still cannot use it to compromise the logs in previous communications sessions.

### 3.3. Procedure of the Proposed Protocol

Communication in the proposed protocol is carried out including 13 phases: initialization phase, device registration phase, SSO registration phase, SSO login phase, SSO password generation phase, user registration phase, log signcryption phase, log verification phase, private block calculation phase, private block verification phase, log unsigncryption phase, public block calculation phase and public block verification phase.

#### 3.3.1. System Initialization Phase

In initialization phase, the attribute authority generates public and secret parameters used in entire system. The attribute authority selects a big number q, and determine the elliptic curve: $E:y^{2}=x^{3}+ax+b\;mod\;q$. It then generates a cyclic group $G_{1}$ and bilinear map $e:G_{1}\times G_{1}\to G_{T}$. g is set as the generator of $G_{1}$. Next, a set of system attributes is determined by $u_{s}=\left\{att_{1},att_{2},\ldots ,att_{x}\right\}$. The authority selects the corresponding random numbers $\left\{Q_{1},Q_{2},\ldots ,Q_{x}\right\}\in_{R}G_{1}$, based on attribute set$\;u_{s}$, and chooses a secure one-way hash function $H:{\left\{0,1\right\}}^{*}\in G_{1}$. It randomly selects $\alpha ,\beta \in_{R}Z_{q}$, and compute $B=g^{\beta }$ and public key $Y=e{\left(g,g\right)}^{\alpha }$. Finally, the authority generates public parameter $PP=\left(g,B,Y,H,Q_{x},e,G_{1},G_{T}\right)$ and secret parameter $MSK=\left(\alpha ,\beta ,u_{s}\right)$. Specific steps of this phase are described in Algorithm 1.
**Algorithm 1:** System initialization.**Input**: Initial parameters. **Output**: PP, MSK. 1:Select a big number q, and determine the elliptic curve: $E:y^{2}=x^{3}+ax+b\;mod\;q$.2:Generate a cyclic group $G_{1}$ and bilinear map $e:G_{1}\times G_{1}\to G_{T}$.3:Set g as the generator of$\;G_{1}$.4:Determine system attribute set $u_{s}=\left\{att_{1},att_{2},\ldots ,att_{x}\right\}$.5:Select $\left\{Q_{1},Q_{2},\ldots ,Q_{x}\right\}\in_{R}G_{1}$, based on attribute$\;u_{s}$.6: Choose a secure one-way hash function $H:{\left\{0,1\right\}}^{*}\in G_{1}$.7: Randomly select $\alpha ,\beta \in_{R}Z_{q}$.8: Compute $B=g^{\beta }$.9:Compute public key $Y=e{\left(g,g\right)}^{\alpha }$.10:Generate $PP=\left(g,B,Y,H,Q_{x},e,G_{1},G_{T}\right)$ and $MSK=\left(\alpha ,\beta ,u_{s}\right)$.

#### 3.3.2. SSO Registration Phase

In this phase, the user $U_{i}$ uses a smart card to register with the SSO server for obtaining multiple services. SSO registration procedure is provided in Algorithm 2 as follows. The user $U_{i}$ enters $SID_{i}$ and $SPW_{i}$ into smart card. The smart card generates a random number $r_{i}$, and computes $A_{i}=H\left(SPW_{i}\right)⊕H\left(r_{i}∥SID_{i}\right)$. The SSO sever then stores $r_{i}$ and $A_{i}$.
**Algorithm2:** SSO registration.**Input**: $SID_{i}$, $SPW_{i}$. **Output**: $r_{i}$, $A_{i}$. 1:$U_{i}$ enters $\;SID_{i}$ and $SPW_{i}$ into smart card.2:Smart card generates $r_{i}$.3:Smart card computes $A_{i}=H\left(SPW_{i}\right)⊕H\left(r_{i}∥SID_{i}\right)$.4:SSO sever stores $r_{i}$ and $A_{i}$.

#### 3.3.3. SSO Login Phase

The user $U_{i}$ enters $SID_{i}$ and $SPW_{i}$ into SSO sever for verifying his/her legitimacy. The user $U_{i}$ enters $SID_{i}$ and $SPW_{i}$ into the SSO server. The SSO server computes $A_{i}{}^{\prime }=H\left(SPW_{i}\right)⊕H\left(r_{i}∥SID_{i}\right)$. It then compares $A_{i}^{\prime }$ and $A_{i}$, in order to verify legitimacy of the user $U_{i}$. Procedure of this phase is presented by Algorithm 3.
**Algorithm 3:** SSO login.**Input**: $SID_{i}$, $SPW_{i}$. **Output**: True or False. 1:$U_{i}$ enters $SID_{i}$ and $SPW_{i}$.2:SSO server computes $A_{i}{}^{\prime }=H\left(SPW_{i}\right)⊕H\left(r_{i}∥SID_{i}\right)$.3:SSO server compares $A_{i}{}^{\prime }$ and $A_{i}$.4:   **if** above check holds, **then** output True, and confirm legitimacy of $U_{i}$.5:   **otherwise**, output False, and terminate the login.

#### 3.3.4. SSO Password Generation Phase

The use $U_{i}$ enters $SID_{i}$, $SPW_{i}$ and $ID_{i}$ so that the server can generate an SSO password. In this way, the user $U_{i}$ can obtain services from multiple servers using this single password. The user $U_{i}$ enters $SID_{i}$, $SPW_{i}$ and $ID_{i}$ into SSO server. The SSO server generates $PW_{i}$ from $SID_{i}$, $SPW_{i}$ and $ID_{i}$. Procedure of this phase is described by Algorithm 4.
**Algorithm 4:** SSO password generation.**Input**: $SID_{i},SPW_{i},ID_{i}$. **Output**: $PW_{i}$. 1:$U_{i}$ enters $SID_{i}$, $SPW_{i}$ and $ID_{i}$ into SSO server.2:SSO server generates $PW_{i}=SSOgen(SID_{i},SPW_{i}$,$ID_{i})$.

#### 3.3.5. Device Registration Phase

In this phase, the $sensor_{i}$ registers with the attribute authority for further communication. The sensor and the authority perform necessary steps for device registration, as presented in Algorithm 5. The $sensor_{i}$ sends $DID_{i}$ to the attribute authority. The authority verifies $DID_{i}$, then sends public parameter PP to the $sensor_{i}$.
**Algorithm 5:** Device registration.**Input**: $DID_{i}$. **Output**: PP. 1:$Sensor_{i}$ sends $DID_{i}$ to attribute authority.2:Attribute authority verifies $DID_{i}$.3:Attribute authority sends PP to $sensor_{i}$.

#### 3.3.6. User Registration Phase

The user $U_{i}$ uses his/her identity $ID_{i}$ to register and obtain the attribute private key from the attribute authority. This procedure is performed by the attribute authority, as specified in Algorithm 6. The user $U_{i}$ first sends his/her identity $ID_{i}$ to the attribute authority. Upon the received message, the attribute authority verifies $ID_{i}$. It then randomly chooses $t_{ID_{i}}\in_{R}Z_{q}$, and uses g,α,β, and $t_{ID_{i}}$ to compute $K^{i}$, $L^{i}=g^{t_{ID_{i}}}$ and $K_{j}^{i}=Q_{j}^{t_{ID_{i}}}\left(\forall \;j\;\in x\right)$. The authority generates attribute private key $SK_{ID_{i}}=\;$($K^{i},L^{i},K_{j}^{i}$), and sends $SK_{ID_{i}}$ to the user $U_{i}$.
**Algorithm 6:** User registration.**Input**: $ID_{i},PP,MSK$. **Output**: $SK_{ID_{i}}$. 1:Receive $ID_{i}$ from $U_{i}$.2:Verify $ID_{i}$.3:Choose $t_{ID_{i}}\in_{R}Z_{q}$.4:Compute $K^{i}=g^{\alpha +\left(\beta t_{ID_{i}}\right)}$.5:Compute $L^{i}=g^{t_{ID_{i}}}$.6:Compute $K_{j}^{i}=Q_{j}^{t_{ID_{i}}}\left(\forall \;j\;\in x\right)$.7:Send $SK_{ID_{i}}=\;$($K^{i},L^{i},K_{j}^{i}$) to $U_{i}$.

#### 3.3.7. Log Signcryption Phase

In this phase, the $sensor_{i}$ is allowed to signcrypt the log, based on attribute-based access policy, Boolean formula BF and public parameters PP. The $sensor_{i}$ performs specific steps in Algorithm 7 for the log signcryption procedure. It sets LSSS matrix $m_{e}\;$by Boolean formula BF, and randomly generates random number $r_{j}\in_{R}Z_{q}\left(\forall \;j\;\in x\right)$ and a secret vector ${\vec{v}}_{e}$ composed by secret signcryption key s and j attributes. Then, the $sensor_{i}$ uses matrix $m_{e}$, vector $\;{\vec{v}}_{e}$, signcryption key s and parameter g to compute $\lambda_{e}=m_{e}{\vec{v}}_{e}$ and $C^{\prime }=g^{s}$. Parameters B, $r_{j}$, g and $\lambda_{e}$ are used to compute $C_{j}=g^{\beta \lambda_{e}}Q_{j}{}^{-r_{j}}\left(\forall \;j\;\in x\right)$ and $D_{j}=g^{r_{j}}\left(\forall \;j\;\in x\right)$. Next, the $sensor_{i}$ computes log ciphertext $C=MY^{s}$, its hash value h=H(C) and signature $\sigma_{CT}=h^{Y_{.\;x}s}$. Thereafter, ECDSA signature $\sigma_{IoT}=ECDSA\left(\sigma_{CT},IP_{IoT},t_{IoT}\right)$ is derived. The $sensor_{i}$ generates ciphertext $CT=\left(\sigma_{CT},C,C_{j},C^{\prime },D_{j},m_{e},IP_{IoT},t_{CT}\right)$, and then sends it to the gateway. Finally, $\delta_{IoT}=\left(\sigma_{IoT},IP_{IoT},t_{IoT}\right)$ is stored by the $sensor_{i}$.
**Algorithm7:** Log signcryption.**Input**: BF,PP,M. **Output**: $CT,\delta_{IoT}$. 1:Set LSSS matrix $m_{e}\;$by BF.2:Randomly generate $r_{j}\in_{R}Z_{q}\left(\forall \;j\;\in x\right)$.3:Generate ${\vec{v}}_{e}=\left[\begin{array}{c} s\\ \begin{array}{c} att_{1}\\ att_{2}\\ \begin{array}{c} \vdots \\ att_{j} \end{array} \end{array} \end{array}\right]\in_{R}Z_{q}\left(\forall \;j\;\in x\right)$.4:Use $m_{e}$ and ${\vec{v}}_{e}$ to compute $\lambda_{e}=m_{e}{\vec{v}}_{e}$.5:Use s and g to compute$\;C^{\prime }=g^{s}$.6:Use B, $r_{j}$ and $\lambda_{e}$ to compute $C_{j}=g^{\beta \lambda_{e}}Q_{j}{}^{-r_{j}}\left(\forall \;j\;\in x\right)$.7:Use $r_{j}$ and g to compute $D_{j}=g^{r_{j}}\left(\forall \;j\;\in x\right)$.8:Compute $C=MY^{s}$.9:Compute h=H(C).10:Compute $\sigma_{CT}=h^{Y_{.\;x}s}$.11:Perform $\sigma_{IoT}=ECDSA\left(\sigma_{CT},IP_{IoT},t_{IoT}\right).$12:Send $CT=\left(\sigma_{CT}\;,C,C_{j},C^{\prime },D_{j},m_{e},IP_{IoT},t_{CT}\right)with\left(m_{e},\rho \left(j\right)\right)$ to gateway.13:Store $\delta_{IoT}=\left(\sigma_{IoT},IP_{IoT},t_{IoT}\right)$.

#### 3.3.8. Log Verification Phase

The gateway verifies the validity of the signatures and the ciphertext CT, based on public parameters PP. If the verifications are valid, the gateway will send it to the blockchain server. This phase is performed by the gateway with Algorithm 8. The gateway checks $e\left(h,Y_{.x}C^{\prime }\right)≟e\left(\sigma ,g\right)$ and $ECDSA\left(\delta_{IoT},\sigma_{CT}\right)$. If the checks hold, it sends ciphertext CT to the blockchain server. The ciphertext is then stored at cluster storage. The gateway computes signature $\sigma_{GW}=ECDSA\left(\sigma_{CT},\sigma_{IoT},IP_{GW},t_{GW}\right)$, sets $\delta_{GW}=\left(\sigma_{GW},IP_{GW},t_{GW}\right)$, and stores $\delta_{GW}$.
**Algorithm 8:** Log Verification.**Input**: $CT,\delta_{IoT},PP$. **Output**: $\delta_{GW}$. 1:$\mathrm{Ve}rify:\;e\left(h,Y_{.\;x}C^{\prime }\right)≟e\left(\sigma_{CT},g\right)$.2:$Verify_{ECDSA}\left(\delta_{IoT},\sigma_{CT}\right)$.3:   **if** above checks hold, **then** continue with step 5.4:   **otherwise**, terminate the session.5: Send CT to blockchain server, CT is then stored in cluster storage.5:Compute signature $\sigma_{GW}=ECDSA\left(\sigma_{CT},\sigma_{IoT},IP_{GW},t_{GW}\right)$.6:Store $\delta_{GW}=\left(\sigma_{GW},IP_{GW},t_{GW}\right)$.

#### 3.3.9. Log Unsigncryption Phase

In log unsigncryption phase, the user $U_{i}$ is allowed to unsigncrypt the ciphertext CT to view the log M, using private key $SK_{ID_{i}}$ (with appropriate attributes) and public parameter PP. The user $U_{i}$ uses Algorithm 9 to complete this procedure. Parameter $w_{i}\;$is first restored from the matrix $m_{e}$ with appropriate attributes. The user $U_{i}$ then uses parameters $C,C^{\prime },D_{j}$ and private key$\;SK_{ID_{i}}=$($K^{i},L^{i},K_{j}^{i}$) to compute decryption key $Y^{s}$. Value h=H(C) is computed for checking $e\left(h,Y_{.x}C^{\prime }\right)≟e\left(\sigma ,g\right)$ based on parameters g, Y, C and $C^{\prime }$. At last, the user $U_{i}$ uses $Y^{s}$ to decrypt log ciphertext and obtain the log by $M=C.Y^{s}{}^{-1}$.
**Algorithm 9:** Log unsigncryption.**Input**: $CT,SK_{ID_{i}},PP$. **Output**: M. 1:Restores $w_{i}$ from $m_{e}$ with appropriate attributes: $\left[\begin{array}{c} 1\\ 0\\ \begin{array}{c} \vdots \\ 0 \end{array} \end{array}\right]=m_{e}w_{j}$.2:Use $C,C^{\prime },D_{j}$ and$\;SK_{ID_{i}}$ to compute decryption key: $Y^{s}=e{\left(g,g\right)}^{s\alpha }=\frac{e\left(C^{\prime },K^{i}\right)}{\Pi_{j\in x}{\left(e\left(C_{j},L^{i}\right)e\left(D_{j}K_{j}^{i}\right)\right)}^{w_{j}}}$.3:Compute h=H(C).4:Use g, Y, C and $C^{\prime }$ to $\mathrm{Ve}rify:\;e\left(h,Y_{.\;x}C^{\prime }\right)≟e\left(\sigma_{CT},g\right)$.5:   **if** above check holds, **then** continue with step 7.6:   **otherwise**, terminate the session.7:Use $Y^{s}$ to decrypt C and obtain $M=C.Y^{s}{}^{-1}$.

#### 3.3.10. Private Block Calculation Phase

Based on the ciphertext CT and some other information, the blockchain server calculates private block data, then writes it to the blockchain. At first, the server retrieves previous hash from the private blockchain, and verifies ECDSA signature $\sigma_{GW}$. It computes signature $\sigma_{Srv}=ECDSA\left(\sigma_{CT},\sigma_{GW},IP_{Srv},t_{Srv}\right)$, and sets $\delta_{Srv}=\left(\sigma_{Srv},IP_{Srv},t_{Srv}\right)$ and $\delta_{CT}=\left(\sigma_{CT},IP_{Srv},t_{CT}\right)$. The initial Nonce value is set as 0. The server then iteratively compute $H\left(Nonce∥PreviousHash∥\delta_{Srv}∥\delta_{GW}∥\delta_{IoT}∥\delta_{CT}∥OptionalFields∥OtherFields\right)$, which must be smaller than the Difficulty level. It sets Nonce=Nonce+1 if above check does not hold, and re-compute the hash. The computing is completed if above condition holds. Block data $PriB=(\delta_{Srv},\delta_{GW},\delta_{IoT},\delta_{CT},Nonce,Difficulty,Optional\;Fields$ is generated and written to the private blockchain. Finally, the server receives a corresponding block number $PriBlockNo_{n}$. Private blockchain calculation procedure is further specified in Algorithm 10.

#### 3.3.11. Private Block Verification Phase

In this phase, the user $U_{i}$ verifies the validity of the private block PriB. Specific steps of private block verification are performed by the user $U_{i}$ with Algorithm 11. The user $U_{i}$ verifies whether $H\left(Nonce∥PreviousHash∥\delta_{Srv}∥\delta_{GW}∥\delta_{IoT}∥\delta_{CT}∥OptionalFields∥OtherFields\right)\;<Difficulty$. The validity of ECDSA signatures $\left(\delta_{IoT},\sigma_{CT}\right)$, $\left(\delta_{GW},\sigma_{CT}\right)$ and $\left(\delta_{Srv},\sigma_{CT}\right)$, and $e\left(h,Y_{.x}C^{\prime }\right)≟e\left(\sigma_{CT},g\right)$ are also verified. The system outputs True if above verifications hold, otherwise outputs False.
**Algorithm 10:** Private block calculation.**Input**: $CT,\delta_{IoT},\delta_{GW},PreviousHash,OptionalFields,OtherFields$. **Output**: PriB. 1:Retrieve previous hash from private blockchain.2:${\mathrm{Verify}}_{ECDSA}\left(\delta_{GW},\sigma_{CT}\right)$.3:   **if** above check holds, **then** continue with step 5.4:   **otherwise**, terminate the session.5:Compute $\sigma_{Srv}=ECDSA\left(\sigma_{CT},\sigma_{GW},IP_{Srv},t_{Srv}\right)$.6:Set $\delta_{Srv}=\left(\sigma_{Srv},IP_{Srv},t_{Srv}\right)$.7:Set $\delta_{CT}=\left(\sigma_{CT},IP_{Srv},t_{CT}\right)$.8:Set initial Nonce value as 0.9:    while $H\left(Nonce∥\mathrm{PreviousHash}∥\delta_{Srv}∥\delta_{GW}∥\delta_{IoT}∥\delta_{CT}∥OptionalFields∥OtherFields\right)<Difficulty$ **do**.10:     Nonce=Nonce+1.11:end while.12:Generate $PriB=\left(\delta_{Srv},\delta_{GW},\delta_{IoT},\delta_{CT},Nonce,Difficulty,Optional\;Fields\right)$.13:Write PriB to private blockchain.14:Receive $PriBlockNo_{n}$ from private blockchain.**Algorithm 11:** Private block verification.**Input**: CT,PriB. **Output**: True or False. 1:Verify if: $H\left(Nonce∥PreviousHash∥\delta_{Srv}∥\delta_{GW}∥\delta_{IoT}∥\delta_{CT}∥OptionalFields∥OtherFields\right)\;<Difficulty$.2:$Verify_{ECDSA}\left(\delta_{IoT},\sigma_{CT}\right)$.3:$Verify_{ECDSA}\left(\delta_{GW},\sigma_{CT}\right)$.4:$Verify_{ECDSA}\left(\delta_{Srv},\sigma_{CT}\right)$.5:$Verify:\;e\left(h,Y_{.x}C^{\prime }\right)≟e\left(\sigma ,g\right)$.6:   **if** verifications hold, **then** output True.7:   **otherwise**, output False.

#### 3.3.12. Public Block Calculation Phase

The blockchain sever computes batch signature from multiple signatures, and write it to public blockchain. In this. way, credibility of the signatures is enhanced with immutability feature. The procedure is performed by the blockchain server with Algorithm 12 as follows. The server retrieves block number $PriBlockNo_{n}$ from the corresponding block $PriB_{n}$, and multiple signatures $\sigma_{CT_{n}}$ from multiple private blocks $PriB_{n}$. Batch signature $BSig=\sigma_{CT_{1}}\sigma_{CT_{2}}\ldots \sigma_{CT_{n}}$ is computed to generate public block $PubB=\left(PriBlockNo_{n},BSig,Optional\;Fields\right)$. Next, the server writes PubB to the public blockchain, and receives the corresponding block number $PubBlockNo_{n}$.

#### 3.3.13. Public Block Verification Phase

In this phase, the user $U_{i}$ verifies the batch verification of data recorded in public blockchain, based on $CT_{n},PubB$ and PP. Algorithm 13 is performed by the user $U_{i}$ to complete this procedure. The user $U_{i}$ confirms if $PriBlockNo_{n}$ is available on the private chain, then obtains the corresponding blocks $PriB_{n}$. Next, the user $U_{i}$ verifies whether BSig matches the signatures in the private blocks $PriB_{n}$. Value $h_{n}=H\left(C_{n}\right)$ is computed based on n log ciphertext $C_{n}$. Finally. the user $U_{i}$ verifies the validity of the batch signature: $e\left(h_{n},\;Y_{.x}{}_{n}C_{n}^{\prime }\right)≟e\left(BSig,g_{n}\right)$. The system outputs True, meaning the verification is successful if the check holds, otherwise outputs False.
**Algorithm 12:** Public block calculation.**Input**: $PriBlockNo_{n}$. **Output**: PubB. 1:Retrieve $PriBlockNo_{n}$ from corresponding block $PriB_{n}$.2:Retrieve $\sigma_{CT_{n}}$ from $PriB_{n}$.3:Compute $BSig=\sigma_{CT_{1}}\sigma_{CT_{2}}\ldots \sigma_{CT_{n}}$.4:Generate $PubB=\left(PriBlockNo_{n},BSig,Optional\;Fields\right)$.5:Write PubB to public blockchain.6:Receive $PubBlockNo_{n}$ from public blockchain.**Algorithm 13:** Public block verification.**Input**: $PubB,CT_{n},PP$. **Output**: True or False. 1:Confirm if $PriBlockNo_{n}$ is available on private blockchain.2:$Verify:BSig≟\sigma_{CT_{1}}\sigma_{CT_{2}}\ldots \sigma_{CT_{n}}$.3:   **if** above check holds, **then** continue with step 6.4:   **otherwise**, terminate current session.5:Compute $h_{n}=H\left(C_{n}\right)$, based on n log ciphertext $C_{n}$.6:$Verify:e\left(h_{i},\;Y_{.x}{}_{n}C_{n}^{\prime }\right)≟e\left(BSig,\;g_{n}\right)$.7:   **if** verification holds, **then** output True.8:   **otherwise**, output False.

## 4. Security Analysis

In this section, we use AVISPA toolset and GNY logic to verify security correctness of the proposed protocol. In addition, we prove that our protocol meets various security requirements based on the semantic proof.

### 4.1. Protocol Simulation Using AVISPA Toolset

We verify the security properties of the proposed protocol by employing Automated Validation of Internet Security Protocols and Applications (AVISPA) [59]. AVISPA tool uses HLPSL [60] as its formal language, and integrates different back-ends in the verification techniques. The back-ends includes On-the-fly Model-Checker (OFMC), Constraint Logic based Attack Searcher (CL-AtSe), SAT-based ModelChecker (SATMC), and Tree Automata based on automatic approximations for the analysis of security protocols (TA4SP). However, the SATMC and TA4SP back-ends are not frequently used since they cannot verify the protocols using algebraic properties of modular exponentiation and XOR operator. In this simulation, AVISPA tool is integrated with Security Protocol Animator (SPAN) for providing a user-friendly application interface.

In our protocol, single signature verifications conducted by the gateway, the user and the blockchain sever are identical. We therefore only simulate the verification of the user in the log unsigncryption phase. Moreover, since the gateway and the sever just merely receives and verifies the signature, and they do not make any changes to the signature, we assume that the user directly receives signature from the sensor. We use the similar arguments of single signature verification for verifying the correctness of the batch signature.

**Figure 3:**
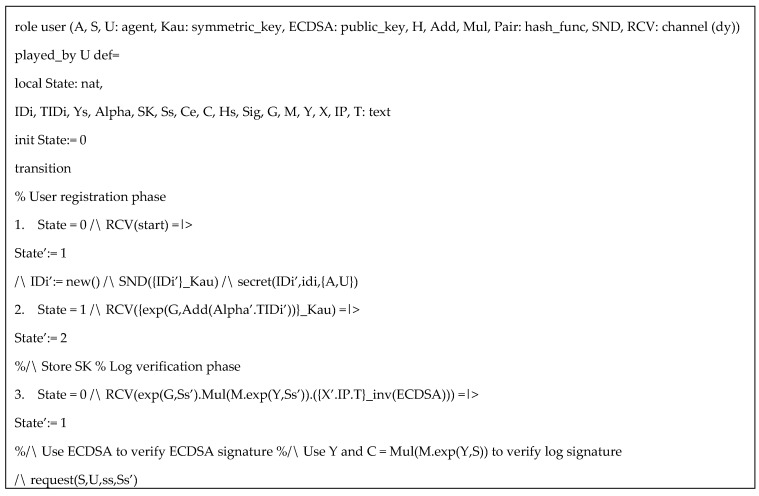

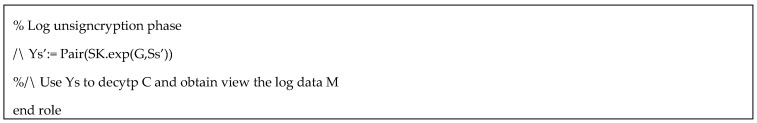
HLPLS specification of user role.

**Figure 4:**
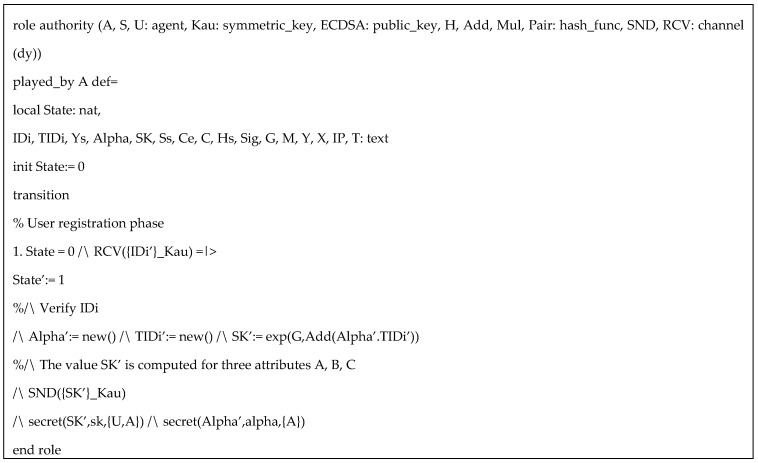
HLPLS specification of attribute authority role.

**Figure 5:**
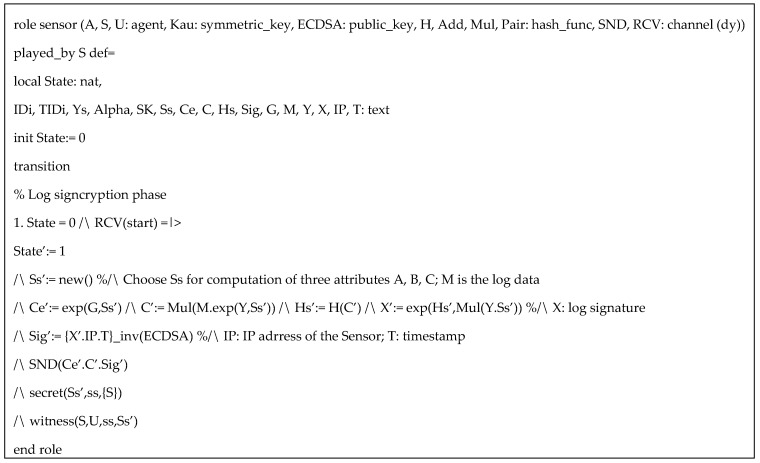
HLPLS specification of sensor role.

HLPLS codes in the simulation was specified with three roles: authority (*A*), sensor (*S*), and user (*U*). The symmetric key *Kau* is used to protect the information in user registration phase. Based on our protocol, $\left(\alpha ,\beta ,u_{s}\right)$ are secret keys of the authority, but for simplicity of the simulation, we only include α. Since AVISPA only support three types of operators (concatenation, exclusive or, and exponentiation), the multiplication and paring function can be performed as hash functions. We also assume ECDSA and inv(ECDSA) are public key and private key respectively, for performing ECDSA algorithms. Specific HLPSL specifications of the user, the authority, and the sensor are provided in Figure 3, Figure 4 and Figure 5 respectively.

**Figure 6:**
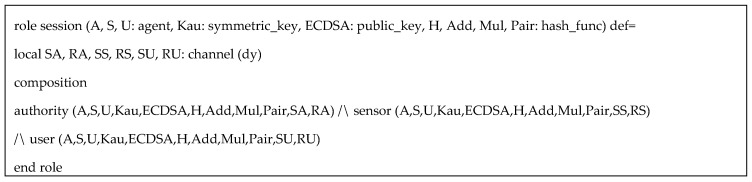
HLPLS specification of session role.

**Figure 7:**
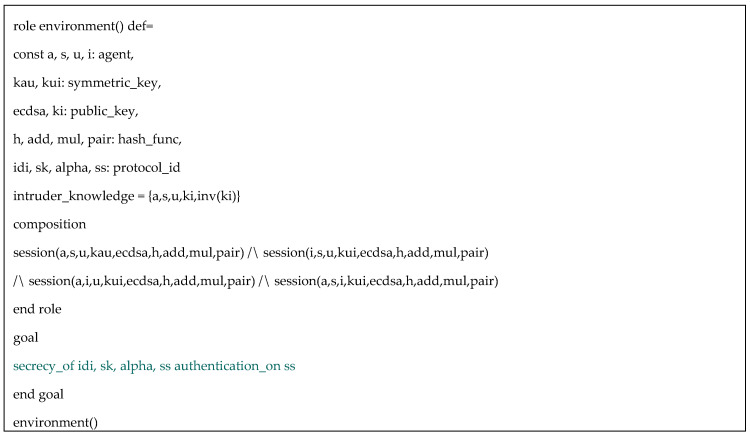
HLPLS specification of environment role.

In addition, Figure 6 provides the specification of the session role where its composition consisting of all main roles is specified. Environment role illuminated in Figure 7 specifies all relevant components within the communication environment including symmetric keys, functions, protocol id, and intruder knowledge. In simulated environment, we can see that the intruder in turn replaces the roles of the authority, the sensor and the user in respective sessions in which, he/she attempts to compromise the simulated system. We consider four secrecy goals and one authentication goal described in the following:

- “*secrecy_of idi*” represents the identity $ID_{i}$ that the user uses to register with the authority via a secure channel, it is kept secret to the user and the authority.
- “*secrecy_of sk*” represents $SK_{ID_{i}}$ that the authority sends to the user, it is also kept secret to the user and the authority.
- “*secrecy_of alpha*” represents the secret value α, it is kept secret to the authority.
- “*secrecy_of ss*” represents the secret key s, it is kept secure to the sensor.
- “*authentication_on ss*”: the user authenticates the sensor on s.

**Figure 8:**
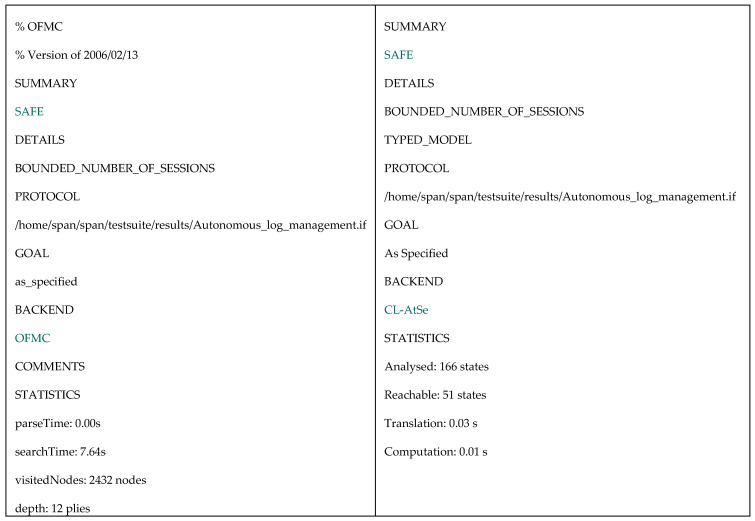
Verification results using OFMC and CL-AtSe backends.

After defining certain communication sessions in environment role, we execute the tool to check the security correctness. The results of OFMC backend and CL-AtSe backend are shown in Figure 8. We claim that the proposed protocol is provably secure under AVISPA simulation.

### 4.2. Logical Analysis Using GNY Logic

This sub-section proves security completeness and correctness of our proposed protocol through Gong-Needham-Yahalom (GNY) logic [61]. For our protocol, the analysis consists of two phases in the logic sequence: message freshness verification and message origin verification. Based on GNY logic, the assumptions and logical rules of our protocol are described in Table 2 and Table 3 respectively [1,62].

Main communication of our protocol can be presented in logic as follows.

$$sensor_{i}\;\to \;U_{i}:\;(h^{Y_{.\;x}s}\;,\left(M\right)e{\left(g,g\right)}^{\alpha s},g^{B\lambda_{e}}Q_{j}{}^{-r_{j}},g^{s},g^{r_{j}},m_{e},IP_{IoT},t_{CT})\;sensor_{i}\;\to \;\mathrm{gateway}:\;h^{Y_{.\;x}s}\;,\left(M\right)e{\left(g,g\right)}^{\alpha s},g^{B\lambda_{e}}Q_{j}{}^{-r_{j}},g^{s},g^{r_{j}},m_{e},IP_{IoT},t_{CT})$$

Specific phases and corresponding goals of the protocol are described in the following.

- Phase 1: Message freshness authentication, proving the authenticity of the message.

Goal 1: Other than the authority, only the user $U_{i}$ can read the content of the message transmitted by the $sensor_{i}$. Goal 1 (G1) is described as follows.

$$U_{i}\;\left|\equiv \;\emptyset \;(h^{Y_{.\;x}s}\;,\left(M\right)e{\left(g,g\right)}^{\alpha s},g^{B\lambda_{e}}Q_{j}{}^{-r_{j}},g^{s},g^{r_{j}},m_{e},IP_{IoT},t_{CT}\right)$$

- Phase 2: Message origin authentication, proving that the message is transmitted by the legitimate $sensor_{i}$.

Goal 2: The user $U_{i}$ can verify that only the $sensor_{i}$ can generate the message received by the $U_{i}$. Description of Goal 2 (G2) is as follows.

$$U_{i}\;|\equiv \;sensor_{i}\;|\sim \;(h^{Y_{.\;x}s}\;,\left(M\right)e{\left(g,g\right)}^{\alpha s},g^{B\lambda_{e}}Q_{j}{}^{-r_{j}},g^{s},g^{r_{j}},m_{e},IP_{IoT},t_{CT})$$

*Goal* 3: The gateway can verify that only the $sensor_{i}$ can generate the message received by the gateway. Goal 3 (G3) is described as follows.

$$\mathrm{Gateway}\;|\equiv \;sensor_{i}\;|\sim \;(h^{Y_{.\;x}s}\;,\left(M\right)e{\left(g,g\right)}^{\alpha s},g^{B\lambda_{e}}Q_{j}{}^{-r_{j}},g^{s},g^{r_{j}},m_{e},IP_{IoT},t_{CT})$$

Based on the assumptions and logical rules, we have the protocol achieve above goals as follows.

Since $U_{i}$ knows of the message, we have that.

(1) $$U_{i}⊲\;*(*\sigma_{CT}\;,C,C_{j},C^{\prime },D_{j},m_{e},IP_{IoT},t_{CT}),$$

According to (T_1_), we have that.

(2) $$U_{i}⊲\;(\sigma_{CT}\;,C,C_{j},C^{\prime },D_{j},m_{e},IP_{IoT},t_{CT}),$$

According to (2), (A_1_) and (T_3_), the user $U_{i}$ can compute secret key $Y^{s}$ and use it to decrypt $C=MY^{s}$, we have that.

(3) $$U_{i}⊲\;(h^{Y_{.\;x}s}\;,\left(M\right)e{\left(g,g\right)}^{\alpha s},g^{B\lambda_{e}}Q_{j}{}^{-r_{j}},g^{s},g^{r_{j}},m_{e},IP_{IoT},t_{CT}),$$

According to (3) and (P), we have that.

(4) $$U_{i}\;϶\;h^{Y_{.\;x}s},\;\left(M\right)e{\left(g,g\right)}^{\alpha s},\;g^{B\lambda_{e}}Q_{j}{}^{-r_{j}},\;g^{s},\;g^{r_{j}},\;m_{e},\;IP_{IoT},\;t_{CT},$$

Based on (4), (A_2_) and (A_3_), $h^{Y_{.\;x}s}$ is truly recognizable. Therefore, according to (A_4_) and (R), we have that.

(5) $$U_{i}\;|\equiv \;\emptyset \;(h^{Y_{.\;x}s},\;\left(M\right)e{\left(g,g\right)}^{\alpha s},\;g^{B\lambda_{e}}Q_{j}{}^{-r_{j}},\;g^{s},\;g^{r_{j}},\;m_{e},\;IP_{IoT},\;t_{CT}),$$

Based on (5), (A_5_), (A_6_), (A_7_), and (F) we achieve G1. Due to (6), (8), (A_3_), (A_4_), (A_5_), (A_6_), (A_7_), and (F), G2 is achieved. Using similar arguments of G2, we realize G3. As a result, the proposed protocol realizes all goals G1, G2 and G3.

### 4.3. Semantic Proof

Our proposed protocol provides secure decryption key, signature verification and data integrity, signature unforgeability, data confidentiality, non-repudiation, tamper proof, and perfect forward secrecy. The specific semantic security proof of the protocol is presented in the following.

- ***Secure decryptionkey***: Based on ECDLP, the adversary cannot retrieve the secret values s from $C^{\prime }$. The secret value α is also hidden in $K^{i}$, and $K^{i}$ is even kept secret to the user and the authority only. Therefore, the adversary is not able to obtain $g^{s}$ and $g^{\alpha }$ for computing the decryption key $Y^{s}=e{\left(g,g\right)}^{s\alpha }$. The key is successfully computed only when the legitimate user performs correct pairing operation with appropriate attributes. Thus, we claim the proposed protocol achieves secure decryption key.
- ***Robustverification and data integrity***: In our protocol, the signature of the ciphertext is verified to assure the authenticity of the log. The verification correctness of single signature $\sigma_{CT_{1}}$ is proved as follows.

(6) $$e\left(h,Y_{.x}C^{\prime }\right)\;=\;e\left(h,Y_{.x}g^{s}\right)\;=\;e\left(h,\;g^{Y_{.x}s}\right)\;=\;e\left(h^{Y_{.x}s},\;g\right)\;=\;e\left(\sigma_{CT_{1}},g\right),$$

Suppose there have two signatures for the batch signing, the following equation proves the correctness of batch signature verification (including $\sigma_{CT_{1}}$ and $\sigma_{CT_{2}}$).

(7) $$e\left(h_{1}h_{2},Y_{.x}Y_{.x}C_{1}^{\prime }C_{2}^{\prime }\right)\;=\;e\left(h_{1}h_{2},Y_{.x}g^{s}Y_{.x}g^{s}\right)\;=\;e\left(h_{1}h_{2},\;g^{Y_{.x}s}g^{Y_{.x}s}\right)\;=\;e\left(h_{1}{}^{Y_{.x}s}h_{2}{}^{Y_{.x}s},\;gg\right)\;=\;e\left(\sigma_{CT_{1}}\sigma_{CT_{2}},gg\;\right)\;=\;e\left(BSig,gg\right),$$

Therefore, the signature is verifiably correct. After verifying that the log and its signature is originally sent and signed by the sensor, the integrity is achieved. Thus, the conclusion is established.

- ***Signatureunforgeability***: If the adversary wants to forge the signature $\sigma =h^{Y_{.x}s}$, he must obtain the correct s. However, as stated, the value s is protected by ECDLP. The adversary therefore is not able to compute s for forging signature σ. So, our work achieves signature unforgeability.
- ***Dataconfidentiality***: If the adversary wants to restore the log M from the ciphertext, he/she must obtain $SK_{ID_{i}}=$($K^{i},L^{i},K_{j}^{i}$) and compute $Y^{s}$ to unsigncrypt C. However, only the user who has registered with the authority possesses correct attributes and the key $SK_{ID_{i}}$. As stated, the security of the decryption key $Y^{s}$ is also guaranteed. Moreover, the adversary does not know of $MSK=\left(\alpha ,\beta ,u_{s}\right)$ to compromise the system. Thus, the confidentiality of the logs is fully achieved.
- ***Non-repudiation and tamper resistance***: During the communication in our protocol, private key s is only known to the sensor. Therefore, the sensor cannot repudiate the signature signed by itself. The signature is furthermore uploaded to public blockchain. Once recorded, it is not possible for block data to be altered retroactively. In this way, the signature cannot be tampered with. Therefore, we claim non-repudiation and data tampering resistance in the proposed protocol.
- ***Perfect forward secrecy****:* In log signcryption phase, the sensor chooses the key s to compute the ciphertext and generate the decryption key $Y^{s}$. Since s is a randomly selected, the key $Y^{s}$ is computed as a nonce. Therefore, even though the adversary has obtained the decryption key of the current session, he/she cannot recover keys of the past communication sessions. Thus, perfect forward secrecy is achieved in our protocol.

### 4.4. Comparison with Related Works

We furthermore indicate contributions of this paper by a comparative study of our work and recently published works discussed in Section 1.2. The comparison is described in Table 4. Symbol √ denotes the protocol achieves the corresponding property, and symbol × denotes the property is not provided by the protocol. Besides, symbol -- denotes the property is not available in the protocol. The results show that the proposed protocol satisfies all essential requirements of security and functionality. Especially, only our protocol provides signature chain with evidence legality, which is useful for digital forensic investigations. Public-private blockchain and signcryption method are also not available in all others works except ours. In addition, autonomous model is only introduced in our work, and Hang and Kim [31]’s work.

## 5. Performance Analysis

In this section, we analyze performance of the proposed protocol based on computation cost. Computation times of major cryptographic functions and operations used in the proposed protocol are defined as follows.

- $T_{E}$: Time of performing an exponentiation operation in $G_{1}$.
- $T_{BP}$: Time of performing a bilinear paring operation.
- $T_{H}$: Time of performing a hash function.
- $T_{ECDSA\_Gen}$: Time of performing an ECDSA generation algorithm.
- $T_{ECDSA\_Veri}$: Time of performing an ECDSA verification algorithm.

Due to SSO solution, computation cost of our protocol is independent of the number of servers. Since generating and verifying the ECDSA are performed using private key and public key, we assume their computation costs are similar to asymmetric encryption and decryption algorithms respectively. Suppose finding the *nonce* value in private blockchain (computing the hash) is straightforward. As shown in Table 5, total cost of the proposed protocol is ($5mT_{E}$ + $2m\left(n+1\right)T_{H}$ + $(2mni+5mn+2m)T_{BP}$ + $3mT_{ECDSA\_Gen}$ + $\left(3mn+2m\right)T_{ECDSA\_Veri}$). Especially, the sensors consume only (5$T_{E}$ + $T_{H}$ + $T_{ECDSA\_Gen}$) for signcrypting a single log. 

**Figure 9:**
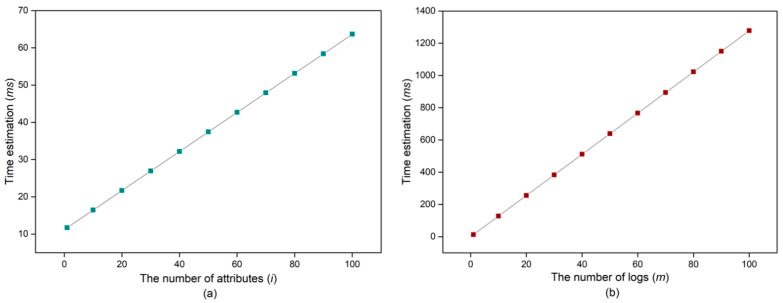
Total computation cost of the proposed protocol: (**a**) n=1 and m=1; (**b**) n=1 and i=3.

Based on the data of Table 5, we further conduct experiments of protocol performance with two scenarios: (a) a single user with i attributes signcrypts a single log, and (b) a single user with certain number of attributes (suppose i=3) signcrypts m logs. In the former scenario (depicted in Figure 9a), the cost is slightly increased when i increases. In the latter scenario (depicted in Figure 9b), when m increases, the cost is significantly increased.

It is observed that our protocol bears a reasonable cost with various components and a lot of functionalities. Moreover, it is important to note that the proposed protocol is designed with ECC small key size, rapid BLS signature, signcryption method, and SSO solution. Our work therefore bears low computation and storage cost, and is well suited for the IoT.

## 6. Implementation

Our implementation simulates log protection of air conditioner $Sensor_{1}$ in the context of a laboratory in Chang Gung University (Taiwan). $Sensor_{1}$ is allowed to signcrypt its log based on an access policy. Attributes generated by the attribute authority including *Chang Gung University, Department of Information Management*, *Professor* and *Student* are set to $att_{1},att_{2},att_{3}$ and $att_{4}$ respectively. User $U_{1}$ attempts to access the log produced by $Sensor_{1}$. The user is able to view the log only if he/she possesses appropriate attributes. We include practical implementation and system construction in the following sub-sections.

### 6.1. Practical Procedure of the Proposed Protocol

**Figure 10:**
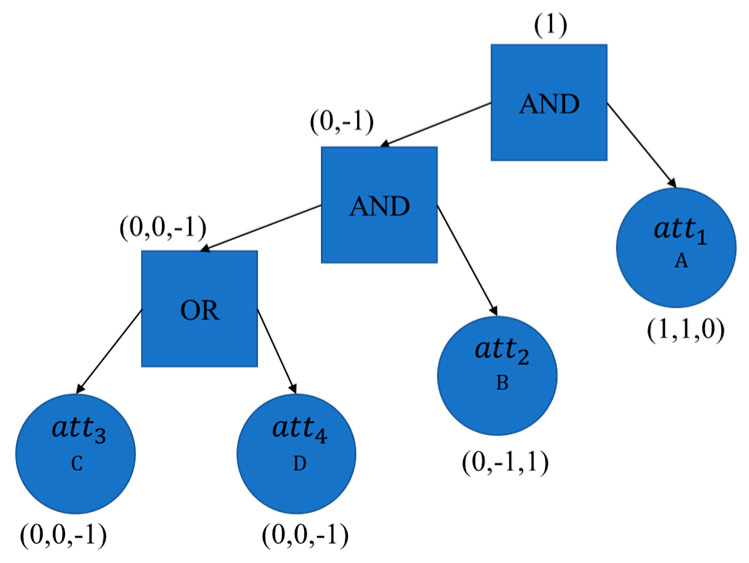
Access policy $BF_{1}$ of $Sensor_{1}$.

Access policy of the sensors are determined with Boolean formula in the beginning. The access policy of $Sensor_{1}$ is illuminated in Figure 10.

At first, the authority initializes the corresponding public parameter $PP=g,B,Y,H,Q_{1},Q_{2},Q_{3},Q_{4},e,G_{1},G_{T}$, and secret parameter $MSK=\alpha ,\beta ,u_{s}$. $Sensor_{1}$ uses its identity $DID_{1}$ to register with the attribute authority and obtains PP. The user $U_{1}$ registers, logs in, and obtains SSO password from the SSO server for accessing specific blockchain server. In this simulation, the user $U_{1}$ possesses three attributes: $att_{1}$, $att_{2}$ and $att_{3}$. In the user registration phase, the attribute authority performs the Algorithm 6 to compute the secret key $SK_{ID_{1}}=\;$($K^{1},L^{1},K_{1}^{1},K_{2}^{1},K_{3}^{1})$, and send it to the user $U_{1}$. Based on attribute-based access policy $BF=“\left(\left(att_{3}\;OR\;att_{4}\right)\;AND\;att_{2}\right)\;AND\;att_{1}”$, $Sensor_{1}$ uses Algorithm 7 to perform signcryption procedure. It then generates $CT_{1}$=($\sigma_{CT_{1}},C^{\prime },C_{1},C_{2},C_{3},D_{1},D_{2},D_{3},C_{CT_{1}},IP_{IoT},t_{CT})\;with\;\left(m_{1},\rho \left(1\right)\right)\left(m_{2},\rho \left(2\right)\right)\left(m_{3},\rho \left(3\right)\right)$ and $\delta_{IoT}^{1}=\left(\sigma_{IoT}^{1},IP_{IoT},t_{IoT}^{1}\right)$. The gateway verifies validity of the ciphertext $CT_{1}$, sends it to the blockchain server, and stores the signature $\delta_{GW}^{1}=\left(\sigma_{GW}^{1},IP_{GW},t_{GW}^{1}\right)$ after performing Algorithm 8. the user $U_{1}$ performs Algorithm 9 to compute the correct key $Y^{s}$ based on the key $SK_{ID_{1}}$ with appropriate attributes. Thus, the user $U_{1}$ is able to unsigncrypt the ciphertext $CT_{1}$ and view the log $M_{1}$.

In private block calculation phase, based on the ciphertext $CT_{1}$ and the previous hash retrieved from the private blockchain, the blockchain server generates block $PriB_{1}=\left(Nonce,\delta_{CT}^{1},\delta_{Srv}^{1},\delta_{GW}^{1},\delta_{IoT}^{1},PreviousHash,Difficulty,OptionalFields,OtherFields\right)$, and upload it to the chain. This procedure is conducted by Algorithm 10. Thereafter, the server obtains the block number $PriBlockNo_{1}$ generated by the private blockchain. For verifying the validity of the block data $PriB_{1}$, the user $U_{1}$ performs Algorithm 11. Suppose we have private block $PriB_{2}$ generated by the similar procedures. In public block calculation phase, the server then calculates batch signature $BSig_{1}=\sigma_{CT_{1}}\sigma_{CT_{2}}$. Block data $PubB_{1}$ is then calculated by $PubB_{1}=\left(BSig_{1},PriBlockNo_{1},PriBlockNo_{2}\right)$, and is uploaded to the public blockchain. Thereafter, the sever can obtain the block number $PubBlockNo_{1}$ generated by the pubic blockchain. The procedure is performed by Algorithm 12. In public block verification phase, the public block data $PubB_{1}$, the ciphertexts $CT_{1},CT_{2}$, and public parameter PP are retrieved. Based on these parameters, the user $U_{1}$ performs Algorithm 13 to verify validity of the uploaded logs.

### 6.2. System Construction

In this sub-section, we construct a system deployment for the proposed protocol. The development environment and system interface of our construction are specifically provided in the following.

#### 6.2.1. Development Environment

**Figure 11:**
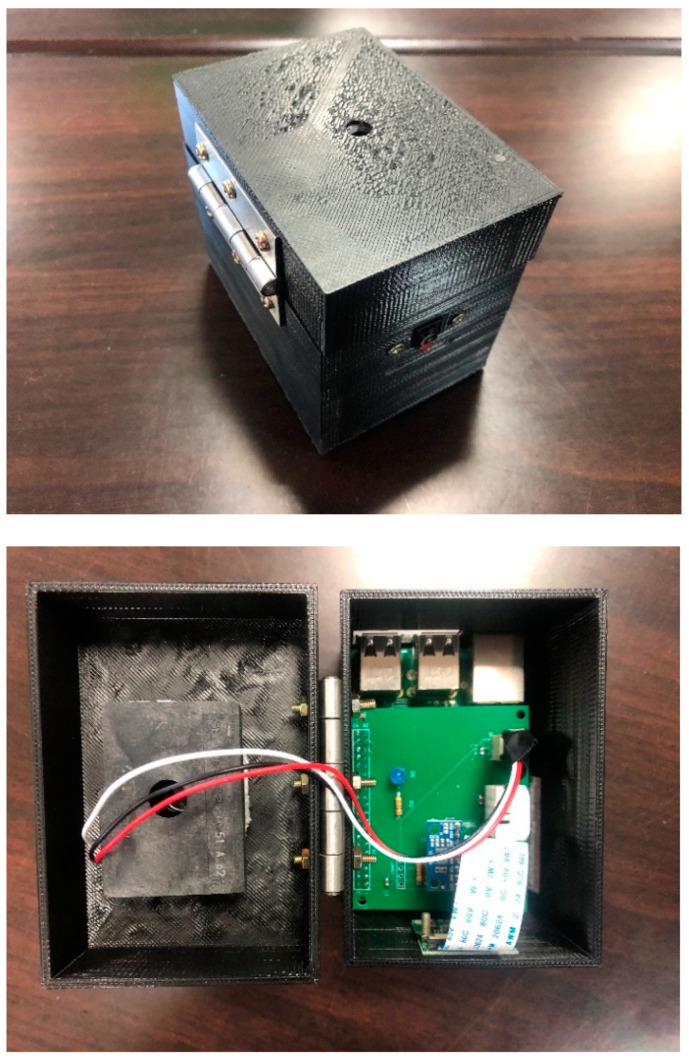
Setting of our implementation.

- Host: We use Ubuntu Server 18.04.02 LTS as the host operating system (OS). The specification includes CPU I7-6820 2.7 GHz, 16 GB RAM memory, and 500 GB hard disk.
- Sensor Configuration: Raspberry Pi is the mainboard used in the system architecture. Raspbian OS is installed with the hardware including Raspberry Pi 3 Model 8 V1.2, CPU ARM Cortex-A53 1.2 GHz Quad Core, 1 GB RAM, and 16 G MicroSD card. Air conditioner sensor YW-51GJ is used as the device of our simulation. The mainboard and the sensor are integrated and assembled in a box so that sensor can contact the ambient air. Overview of our setting is shown in Figure 11.
- Blockchain platform: We employ Hyperledger Fabric v1.0 for private blockchain. This framework provides development foundation, modularity, scalability, and security for the simulated system. We use open source platform of the Ethereum for public blockchain. Smart contract can be fully written and published on Ethereum to develop diversified applications. In this regard, an account with virtual currency is created with Metamask wallet for data transaction. In addition, the contract is written into blockchain through Infura, an Ethereum application programming interface (API).
- Kubernetes: We use Kubernetes as the platform, providing microservices for data management, deployment, and expansion. Kubernetes is compatible to all OS platforms, which provides the proper use of system performance. In our simulation, private blockchain is installed with Kubernetes version 1.13.4.
- Programming language: Languages used in our system includes Golang, HTML and Javascript. Golang library package was developed by Ben Lynn, who developed the library from the original Pairing-Based Cryptography (PBC) written in C language. According to the recommendation of NIST, the selected key length of ECC for security strength is 224-bit type.

#### 6.2.2. System Interface

**Figure 12:**
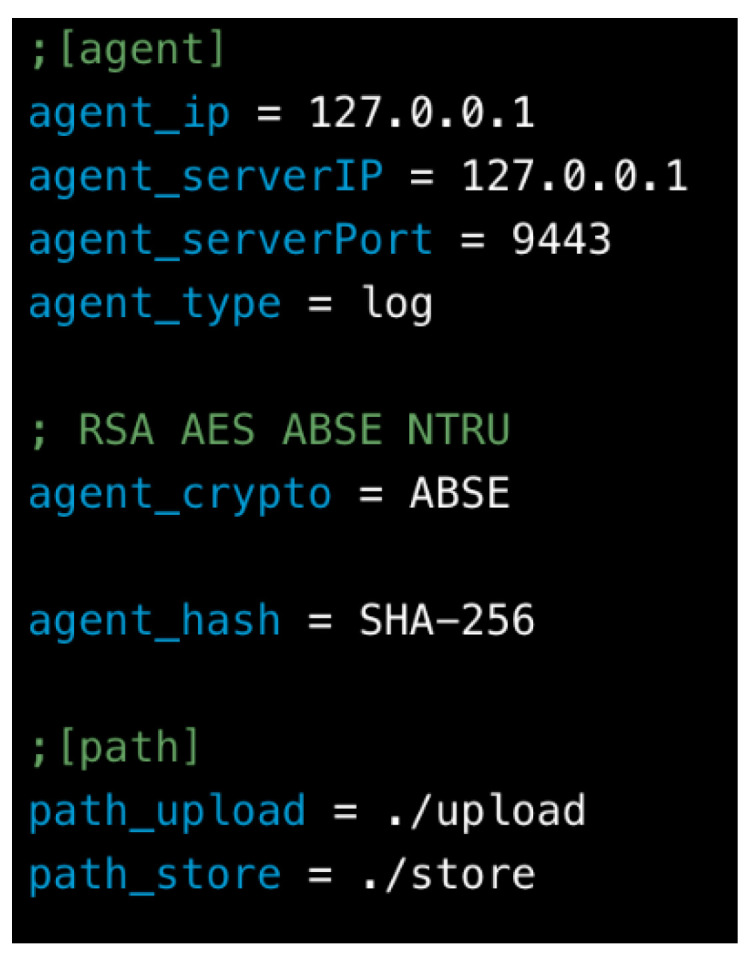
Device configuration.

The device can be configured as an agent with related settings. The configuration file includes agent IP address, server IP address, server port, and agent type. In addition, the cryptographic module supports AES, NTRU, RSA and ABSE, based on the choice of specific cryptosystem. Brief description of device configuration is provided in Figure 12.

**Figure 13:**
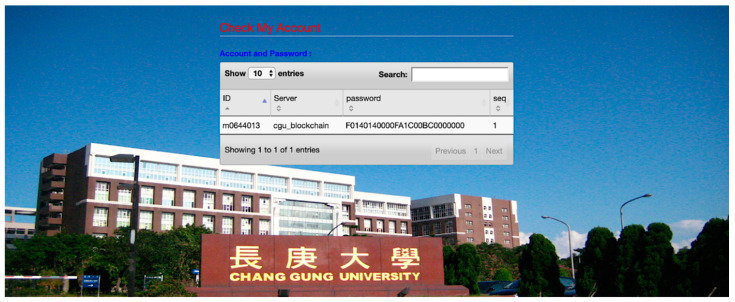
Account generated by SSO server.

The user logs in to the SSO sever using his/her password and smart card. After SSO login, the user has to make another registration with the blockchain sever. To this end, the user enters main identity, password and an additional identity “m0644013” to register with the server of Chang Gung university, named “cgu_blockchain”. SSO password is generated in this registration. Figure 13 shows a registered account with its credential generated by the SSO server for targeted service. Using the account generated by the SSO server, the user can log in to the server “cgu_blockchain”.

**Figure 14:**
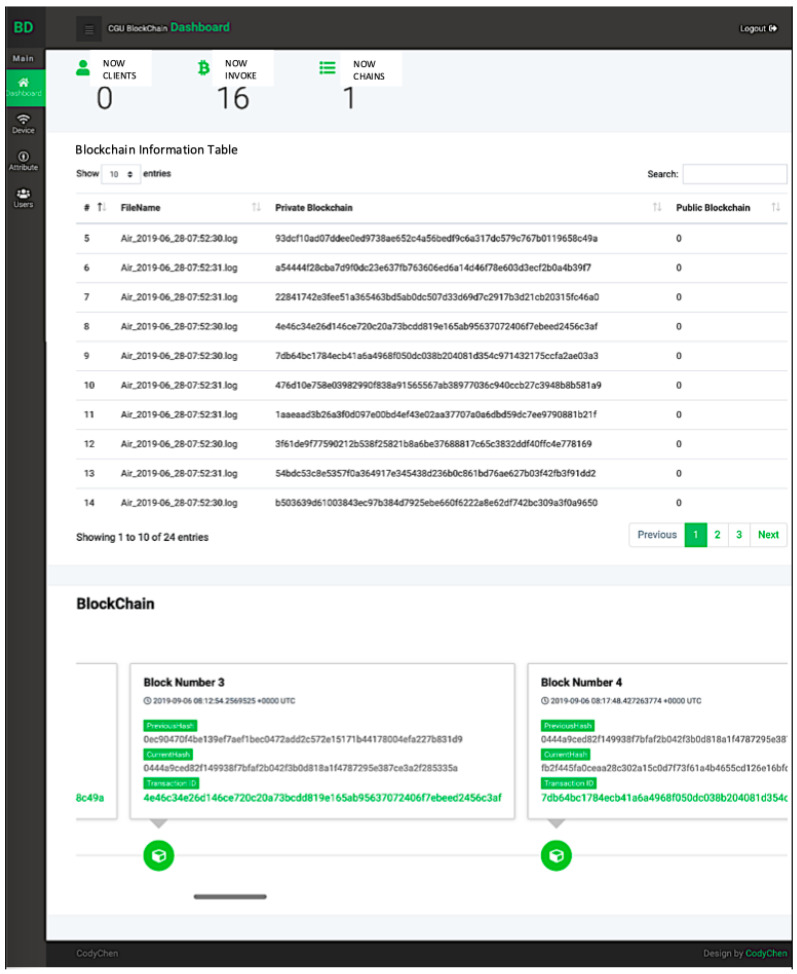
Blockchain server management interface.

**Figure 15:**
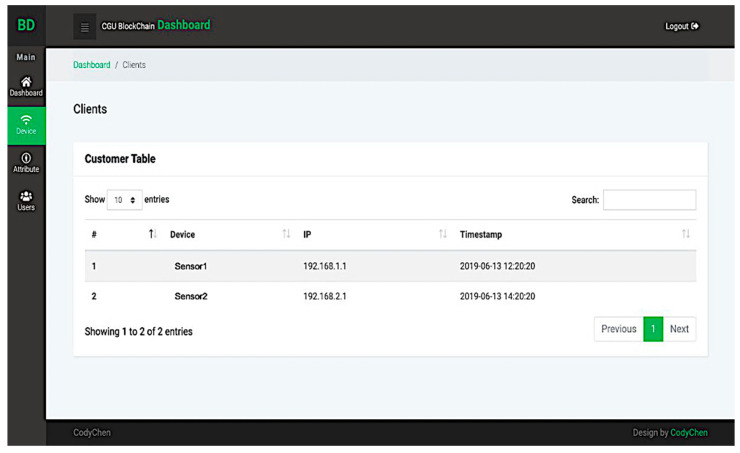
Table of registered devices.

If an administrator enters the system, he/she can see the blockchain status and overall service through a monitoring interface. The interface is described in Figure 14. Table providing information of registered devices is also shown in Figure 15. The devices signcrypt the log and uploads it to blockchain server.

In terms of attribute setting, the attribute authority is allowed to add or delete the attributes, in order to initialize the system. In this way, the authority can determine attributes for specific users, and allows them to obtain their attributes during user registration phase.

**Figure 16:**
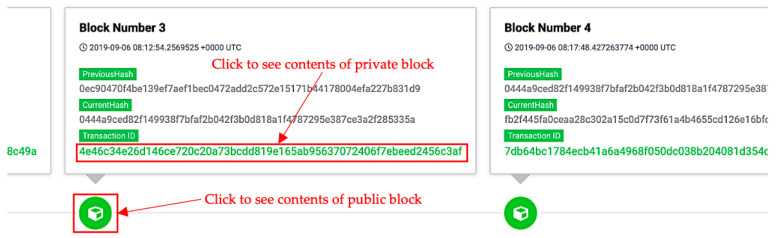
Private blocks within the chain.

**Figure 17:**
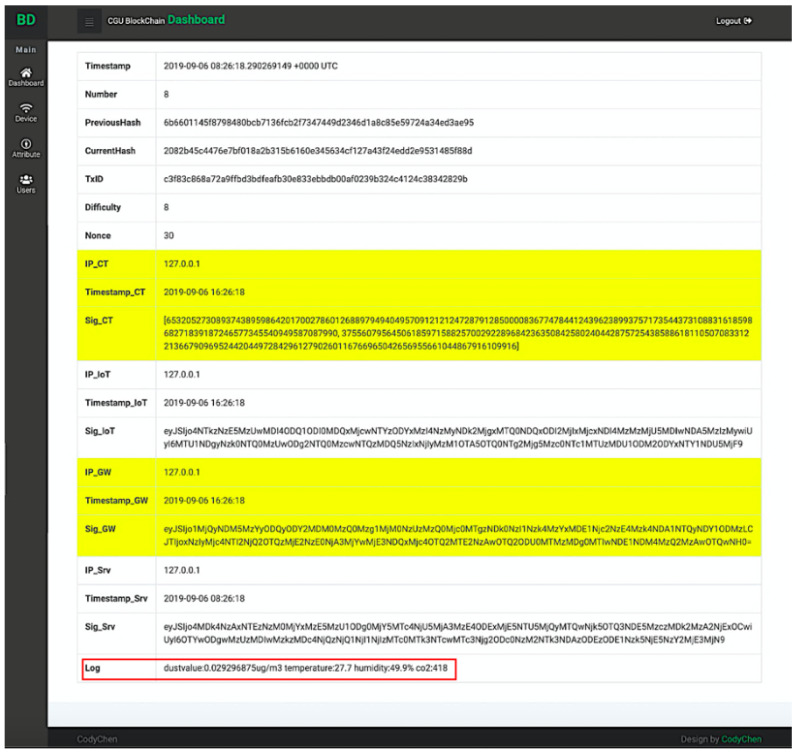
Data stored in a private block.

When the users log in to the system, they can click on the link within private blockchain allocated at the bottom of the monitoring interface (shown in Figure 16) to see block data. The detailed information of private blockchain is provided in Figure 17. In addition, the users (including the administrator and the users who possesses appropriate attributes) can also view the log data at the bottom of this interface. As shown in Figure 17, the log data produced by the sensor includes dust, temperature, humidity, and atmospheric carbon dioxide.

**Figure 18:**
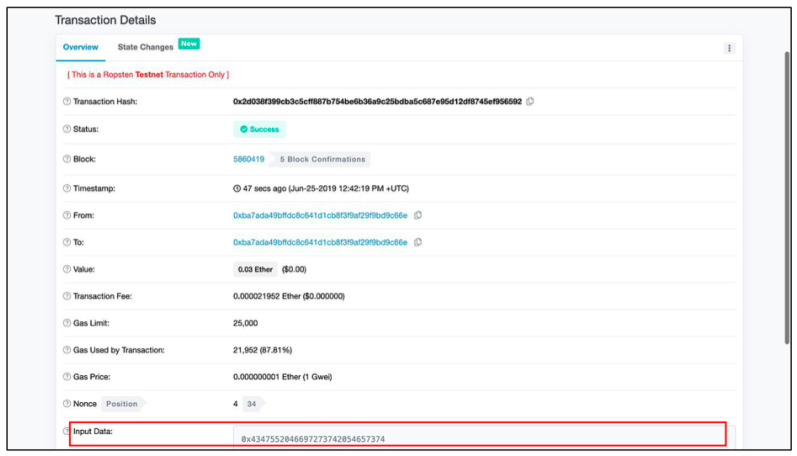
Data stored in a public block.

Furthermore, the user can see detailed information stored in public block by clicking on the icon highlighted in Figure 16. As shown in Figure 18, along with all related information, the data input from private blockchain is available at the bottom of the content table of the public blockchain.

## 7. Conclusions

The logs produced by IoT devices contain important contents and private information, and can be used as evidences for digital forensic investigations. Our work has introduced an autonomous log storage management protocol with blockchain mechanism and access control for the IoT. Various security properties for the logs including robust identity verification, data integrity, non-repudiation, insider attack resistance, and the legality are achieved by the integration of blockchain and signature chain. Moreover, internal security issue has been addressed by fine-grained attribute-based access control. Security analysis demonstrates that our protocol satisfies various security requirements. Our work is well suited for the IoT with a good performance due to elliptic curve short key length, short BLS signature, efficient signcryption method, and multi-server architecture.

In this paper, we propose a protocol with general IoT applications. Domain applications (for instance, WBANs or smart electricity grids) with specific architectures will be considered for our future works with similar mechanisms. Autonomous model would be modified to other ones in which gateways or servers will perform the signscryption. Additionally, protocol performance should also be taken into account, which further improves communication efficiency of low-power devices in the IoT.

## Figures and Tables

**Table 1:** Cryptographic functions and notations used in this paper.

Notations	Description
PP	Public parameters
MSK	Secret parameters
Y	Public key of the authority
α	Secret key of the authority
$C^{\prime }$	Public signcryption key
s	Private signcryption key
M	Log plaintext
C	Log ciphertext
$\sigma_{CT}$	Log signature
$\sigma_{IoT}$	Sensor signature
$\sigma_{GW}$	Gateway signature
$\sigma_{Srv}$	Server signature
t	Timestamp
IP	Internet protocol address
H()	Secure one-way hash function
ECDSA( )	ECDSA signature function
$Verify:{\;}_{ECDSA}\left(\;\right)$	Verifying ECDSA signature function
${\vec{v}}_{e}$	Secret vector
BF	Access policy based on Boolean formula
x	Total number of attributes
$ID_{i}$	Identity of the user

**Table 2:** The assumptions of the proposed protocol.

(A_1_) $U_{i}\;∍\;Y^{s}$: The user $U_{i}$ possesses secret key $Y^{s}$
(A_2_) $sensor_{i}\;∍\;s$: The $sensor_{i}$ possesses private keys s
(A_3_) $U_{i}\;∍\;g^{s}$: The user $U_{i}$ know of public key $g^{s}$
(A_4_) $U_{i}$ |≡ $\emptyset \;\sigma_{CT}$: The user $U_{i}$ believes that $\sigma_{CT}$ is recognizable
(A_5_) $U_{i}$ |≡ # ($m_{e}$): The user $U_{i}$ believes that $m_{e}$ is fresh
(A_6_) $U_{i}$ |≡ # ($IP_{IoT}$): The user $U_{i}$ believes that $IP_{IoT}$ is fresh
(A_7_) $U_{i}$ |≡ # ($t_{CT}$): The user $U_{i}$ believes that timestamp $t_{CT}$ is fresh

**Table 3:** The logical rules of the proposed protocol.

(F) $\frac{U|\equiv \#\left(M\right)}{U\left|\equiv \#\left(M,Y\right),U\right|\equiv \#\left(F\left(M\right)\right)}$: *U* believes message *M* is fresh, which means *U* can believe that any (*M*, *N*) including message *M* is fresh, then *U* believes *F*(*M*), which is computed from message *M*, is also fresh
(P) $\frac{U⊲M}{U∋M}$: *U* can see the message *M*, indicating that *U* really possesses the message *M*
(R) $\frac{U|\equiv \emptyset \left(M\right)}{U\left|\equiv \emptyset \left(M,N\right),\;U\right|\equiv \emptyset \left(F\left(M\right)\right)}$: *U* believes message *M* is recognizable, indicating that *U* can believe that any (*M*, *N*) including message *M* is recognizable, and *U* believes that any *F*(*M*) computed from message *M* is also recognizable)
(T_1_) $\frac{U⊲*M}{U⊲M}$: when *U* obtains a non-original value *M, it means *U* may obtain the original *M*
(T_3_) $\frac{U⊲{\left\{M\right\}}_{K},\;U∋Y}{U⊲M}$: *U* uses secret key *Y* to encrypt, decrypt to obtain message *M*

**Table 4:** Comparison on security and functionality of our work and related works.

	[13]	[29]	[30]	[31]	[32]	[33]	[34]	[35]	Ours
Autonomous model	×	×	×	√	×	×	×	×	√
Signcryption method	×	×	×	×	×	×	×	×	√
Fine-grained access control with ABE	×	×	×	×	√	√	√	√	√
Blockchain mechanism	√	√	√	√	×	√	×	×	√
Integration of private and public blockchain	×	×	×	×	--	×	--	--	√
Signature chain	×	×	×	×	×	×	×	×	√
Evidence legality	×	--	--	×	--	--	--	--	√
Signature unforgeability	√	--	--	--	--	--	--	--	√
Data non-repudiation	√	--	--	√	--	--	--	--	√
Data integrity	√	--	√	√	--	--	--	--	√
Data tampering resistance	√	√	√	×	×	√	×	×	√
Perfect forward secrecy	--	--	--	--	√	√	√	√	√
Protocol simulation using AVISPA/ProVerif	×	×	×	×	×	×	×	×	√
Protocol implementation	×	×	×	√	×	√	×	×	√

**Table 5:** Execution time complexities of the proposed protocol.

	Sensor	Gateway	User	Server
Log signcryption phase	(5$T_{E}$ + $T_{H}$ + $T_{ECDSA\_Gen}$)m	--	--	--
Log verification phase	--	(2$T_{BP}$ + $T_{ECDSA\_Gen}$ + $T_{ECDSA\_Veri}$)m	--	--
Log unsigncryption phase	--	--	(2$iT_{BP}$ + 3$T_{BP}$ + $T_{H}$)mn	--
Private block calculation phase	--	--	--	($T_{ECDSA\_Gen}$ + $T_{ECDSA\_Veri}$ + $T_{H}$)m
Private block verification phase	--	--	($T_{H}$ + 2$T_{BP}$ + 3$T_{ECDSA\_Veri}$)mn	--
Total time complexities	$5mT_{E}$ + $2m\left(n+1\right)T_{H}$ + $(2mni+5mn+2m)T_{BP}$ + $3mT_{ECDSA\_Gen}$ + $\left(3mn+2m\right)T_{ECDSA\_Veri}$			
Total time estimation (ms)	0.5244mni + 3.47346mn + 7.72938m			

m: no. of logs that can be accessed by a single user; n: no. of users; i: no. of attributes; --: not available. Based on [1,63]: $T_{E}$ ≈ 0.72036ms, $T_{BP}$ ≈ 0.2622ms, $T_{H}$ ≈ 0.00069ms, $T_{ECDSA\_Gen}$ ≈ 0.72036ms, and $T_{ECDSA\_Veri}$ ≈ 0.72036ms.

## References

[1] Wong A.K., Hsu C.L., Le T.V., Hsieh M.C., Lin T.W. (2020). Three-Factor Fast Authentication Scheme with Time Bound and User Anonymity for Multi-Server E-Health Systems in 5G-Based Wireless Sensor Networks. Sensors.

[2] Homaei M.H., Salwana E., Shamshirband S. (2019). An Enhanced Distributed Data Aggregation Method in the Internet of Things. Sensors.

[3] Movassaghi S., Abolhasan M., Lipman J., Smith D., Jamalipour A. (2014). Wireless Body Area Networks: A Survey. IEEE Commun. Surv. Tutor..

[4] Guo X., Lin H., Wu Y., Peng M. (2020). A new data clustering strategy for enhancing mutual privacy in healthcare IoT systems. Future Gener. Comput. Syst..

[5] Abdelmoneem R.M., Benslimane A., Shaaban E. (2020). Mobility-aware task scheduling in cloud-Fog IoT-based healthcare architectures. Comput. Netw..

[6] Babar M., Tariq M.U., Jan M.A. (2020). Secure and resilient demand side management engine using machine learning for IoT-enabled smart grid. Sustain. Cities Soc..

[7] Kang L., Chen W., Zheng Z., Li Z., Liang W. (2019). A Novel Debt-Credit Mechanism for Blockchain-Based Data-Trading in Internet of Vehicles. IEEE Internet Things J..

[8] Praveen M., Harini V. NB-IOT based smart car parking system. Proceedings of the 2019 International Conference on Smart Structures and Systems (ICSSS).

[9] Zhang R., Cui S., Zhao C. (2018). Design of a Data Acquisition and Transmission System for Smart Factory Based on NB-IoT..

[10] Yang C.T., Kristiani E., Wang Y.T., Min G., Lai C.H., Jiang W.J. (2020). On construction of a network log management system using ELK Stack with Ceph. J. Supercomput..

[11] Rochim A.F., Aziz M.A., Fauzi A. Design Log Management System of Computer Network Devices Infrastructures Based on ELK Stack. Proceedings of the ICECOS 2019—3rd International Conference on Electrical Engineering and Computer Science.

[12] Di Tosto G., McAlearney A.S., Fareed N., Huerta T.R. (2020). Metrics for Outpatient Portal Use Based on Log. File Analysis: Algorithm Development. J. Med. Internet Res..

[13] Ryu J.H., Sharma P.K., Jo J.H., Park J.H. (2019). A blockchain-based decentralized efficient investigation framework for IoT digital forensics. J. Supercomput..

[14] Harbawi M., Varol A. An improved digital evidence acquisition model for the Internet of Things forensic I: A theoretical framework. Proceedings of the 2017 5th International Symposium on Digital Forensic and Security (ISDFS).

[15] Janjua K., Shah M.A., Almogren A., Khattak H.A., Maple C., Din I.U. (2020). Proactive forensics in IoT: Privacy-aware log-preservation architecture in fog-enabled-cloud using holochain and containerization technologies. Electronics.

[16] Fernández-Caramés T.M., Fraga-Lamas P. (2018). A Review on the Use of Blockchain for the Internet of Things. IEEE Access.

[17] Yuan Y., Wang F. Towards blockchain-based intelligent transportation systems. Proceedings of the 2016 IEEE 19th International Conference on Intelligent Transportation Systems (ITSC).

[18] Gordon W.J., Catalini C. (2018). Blockchain Technology for Healthcare: Facilitating the Transition to Patient-Driven Interoperability. Comput. Struct. Biotechnol. J..

[19] Samaniego M., Jamsrandorj U., Deters R. Blockchain as a Service for IoT. Proceedings of the 2016 IEEE International Conference on Internet of Things (iThings) and IEEE Green Computing and Communications (GreenCom) and IEEE Cyber, Physical and Social Computing (CPSCom) and IEEE Smart Data (SmartData).

[20] Panarello A., Tapas N., Merlino G., Longo F., Puliafito A. (2018). Blockchain and IoT Integration: A Systematic Survey. Sensors.

[21] Queiroz M.M., Wamba S.F. (2019). Blockchain adoption challenges in supply chain: An empirical investigation of the main drivers in India and the USA. Int. J. Inf. Manag..

[22] Wang Y., Singgih M., Wang J., Rit M. (2019). Making sense of blockchain technology: How will it transform supply chains?. Int. J. Product. Econom..

[23] Zyskind G., Nathan O., Pentland A. (2015). Enigma: Decentralized Computation Platform with Guaranteed Privacy. arXiv.

[24] Huang Z., Su X., Zhang Y., Shi C., Zhang H., Xie L. A decentralized solution for IoT data trusted exchange based-on blockchain. Proceedings of the 2017 3rd IEEE International Conference on Computer and Communications (ICCC).

[25] Axon L., Goldsmith M. (2017). PB-PKI: A Privacy-Aware Blockchain-Based PKI..

[26] Kebande V.R., Ray I. A Generic Digital Forensic Investigation Framework for Internet of Things (IoT). Proceedings of the 2016 IEEE 4th International Conference on Future Internet of Things and Cloud (FiCloud).

[27] Perumal S., Norwawi N.M., Raman V. Internet of Things(IoT) digital forensic investigation model: Top.-down forensic approach methodology. Proceedings of the 2015 Fifth International Conference on Digital Information Processing and Communications (ICDIPC).

[28] MacDermott A., Baker T., Shi Q. Iot Forensics: Challenges for the Ioa Era. Proceedings of the 2018 9th IFIP International Conference on New Technologies, Mobility and Security (NTMS).

[29] Taguchi Y., Kanai A., Tanimo S. A Distributed Log. Management Method using a Blockchain Scheme. Proceedings of the 2020 IEEE International Conference on Consumer Electronics (ICCE).

[30] Pourmajidi W., Miranskyy A. Logchain: Blockchain-Assisted Log. Storage. Proceedings of the 2018 IEEE 11th International Conference on Cloud Computing (CLOUD).

[31] Hang L., Kim D.-H. (2019). Design and Implementation of an Integrated IoT Blockchain Platform for Sensing Data Integrity. Sensors.

[32] Li H., Lan C., Fu X., Wang C., Li F., Guo H. (2020). A Secure and Lightweight Fine-Grained Data Sharing Scheme for Mobile Cloud Computing. Sensors.

[33] Zheng H., Shao J., Wei G. (2020). Attribute-based encryption with outsourced decryption in blockchain. Peer-to-Peer Netw. Appl..

[34] Sowjanya K., Dasgupta M. (2020). A ciphertext-policy Attribute based encryption scheme for wireless body area networks based on ECC. J. Inf. Sec. Appl..

[35] Zhong H., Zhou Y., Zhang Q., Xu Y., Cui J. (2020). An efficient and outsourcing-supported attribute-based access control scheme for edge-enabled smart healthcare. Future Gener. Comput. Syst..

[36] Panko R., Bidgoli H. (2004). Digital Signatures and Electronic Signatures. The Internet Encyclopedia.

[37] Nguyen T., Kim K. (2018). A survey about consensus algorithms used in Blockchain. J. Inf. Process. Syst..

[38] Lewko A., Waters B. (2012). New Proof Methods for Attribute-Based Encryption: Achieving Full Security through Selective Techniques. Advances in Cryptology—CRYPTO 2012.

[39] Beimel A. (1996). Secure Schemes for Secret Sharing and Key Distribution.

[40] Shamir A. (1985). Identity-Based Cryptosystems and Signature Schemes. Advances in Cryptology.

[41] Sahai A., Waters B. (2005). Fuzzy Identity-Based Encryption. Advances in Cryptology—EUROCRYPT 2005.

[42] Lai J., Deng R.H., Guan C., Weng J. (2013). Attribute-Based Encryption With Verifiable Outsourced Decryption. IEEE Trans. Inf. Forensics Sec..

[43] Han J., Susilo W., Mu Y., Yan J. (2012). Privacy-Preserving Decentralized Key-Policy Attribute-Based Encryption. IEEE Trans. Parallel Distrib. Syst..

[44] Bethencourt J., Sahai A., Waters B. Ciphertext-Policy Attribute-Based Encryption. Proceedings of the 2007 IEEE Symposium on Security and Privacy (SP’07).

[45] Waters B. (2011). Ciphertext-Policy Attribute-Based Encryption: An. Expressive, Efficient, and Provably Secure Realization. Public Key Cryptography—PKC 2011.

[46] Han J., Susilo W., Mu Y., Zhou J., Au M.H.A. (2015). Improving Privacy and Security in Decentralized Ciphertext-Policy Attribute-Based Encryption. IEEE Trans. Inf. Forensics Sec..

[47] Zheng Y. (1997). Digital signcryption or how to achieve cost(signature & encryption) ≪ cost(signature) + cost(encryption). Advances in Cryptology—CRYPTO ‘97.

[48] Gagné M., Narayan S., Safavi-Naini R. (2010). Threshold Attribute-Based Signcryption. Security and Cryptography for Networks.

[49] Hankerson D., Menezes A., van Tilborg H.C.A., Jajodia S. (2011). Elliptic Curve Discrete Logarithm Problem. Encyclopedia of Cryptography and Security.

[50] Gordon D., van Tilborg H.C.A., Jajodia S. (2011). Discrete Logarithm Problem. Encyclopedia of Cryptography and Security.

[51] Boneh D., Lynn B., Shacham H. (2011). Short Signatures from the Weil Pairing. Advances in Cryptology—ASIACRYPT 2001.

[52] Al-Zubaidie M., Zhang Z., Zhang J. (2019). Efficient and Secure ECDSA Algorithm and its Applications: A Survey. arXiv.

[53] Nakamoto S. (2009). Bitcoin: A Peer-to-Peer Electronic Cash System. Cryptography Mailing List. https://git.dhimmel.com/bitcoin-whitepaper/.

[54] Croman K., Decker C., Eyal I., Gencer A.E., Juels A., Kosba A., Miller A., Saxena P., Shi E., Gün Sirer E. (2016). On Scaling Decentralized Blockchains. Financial Cryptography and Data Security.

[55] Eyal I., Sirer E.G. (2018). Majority is not enough: Bitcoin mining is vulnerable. Commun. ACM.

[56] Henry R., Herzberg A., Kate A. (2018). Blockchain Access Privacy: Challenges and Directions. IEEE Sec. Priv..

[57] Yeow K., Gani A., Ahmad R.W., Rodrigues J.J.P.C., Ko K. (2018). Decentralized Consensus for Edge-Centric Internet of Things: A Review, Taxonomy, and Research Issues. IEEE Access.

[58] Nongbri I., Hadem P., Chettri S. (2018). A Survey on Single Sign-On. Int. J. Creative Res. Thoughts.

[59] Team T.A. Automated Validation of Internet Security Protocols and Applications (AVISPA 1.1). http://www.avispa-project.org.

[60] Von Oheimb D. The high-level protocol specification language HLPSL developed in the EU project AVISPA. Proceedings of the APPSEM 2005 Workshop.

[61] Gong L., Needham R., Yahalom R. Reasoning about belief in cryptographic protocols. Proceedings of the 1990 IEEE Computer Society Symposium on Research in Security and Privacy.

[62] Arshad H., Rasoolzadegan A. (2016). Design of a Secure Authentication and Key Agreement Scheme Preserving User Privacy Usable in Telecare Medicine Information Systems. J. Med. Syst..

[63] Hsu C.L., Le T.V., Hsieh M.C., Tsai K.Y., Lu C.F., Lin T.W. (2020). Three-Factor UCSSO Scheme With Fast Authentication and Privacy Protection for Telecare Medicine Information Systems. IEEE Access.

